# A comprehensive study on the underlying mechanisms of the lipid-lowering effects of Bao Li Er Capsule in hyperlipidemia

**DOI:** 10.1016/j.jtcme.2025.03.006

**Published:** 2025-03-21

**Authors:** Xuelan Fu, Jiehong Xing, Chengjun Yuan, Qingling Liu, Jixiao Zhu, Jinxiang Zeng, Haisha Lu, Huiqing Li, Guoyue Zhong, Jian Liang

**Affiliations:** a Research Center for Chinese Medicine Resources and Ethnic Minority Medicine, Jiangxi University of Chinese Medicine, 1688 Meiling Avenue, Nanchang, 330004, China; b Inner Mongolia Mongolian Medicine Co., Ltd., West Liao River Avenue, Tongliao, 028000, China; c School of Pharmacy, China Pharmaceutical University, 24 Tongjia Street, Nanjing, 211198, China

**Keywords:** Bao Li Er capsule, Network pharmacology, Hyperlipidemia, Lipid-lowering, Antioxidant capacity

## Abstract

**Background and aim:**

A traditional Mongolian formula Bao Li Er capsule (BLEC) has demonstrated excellent therapeutic effect against hyperlipidemia; however, its underlying mechanism of action remains unknown. This study aimed to investigate how BLEC lowers hyperlipidemia (HLP) in rats using network pharmacology, molecular docking, and in vivo experiments.

**Methods and results:**

Active components from BLEC's 21 herbal ingredients were identified using the Traditional Chinese Medicine Systems Pharmacology Database and literature sources. Predicted targets were analyzed using DrugBank and GeneCards databases. Intersection genes were mapped to construct a protein-protein interaction (PPI) network via the STRING database. Molecular docking assessed the binding affinities between core components and key targets. Gene ontology and Kyoto Encyclopedia of Genes and Genomes pathway enrichment analyses were performed using the DAVID database, with findings validated through animal experiments. Network pharmacology identified 268 common targets between BLEC and HLP. Among these, five core components, including quercetin, exhibited strong binding affinity with the key HLP target, PPARG. Animal studies demonstrated that BLEC significantly downregulated PPARG and IL-6 levels, reduced Apo-B100 content, and upregulated ABCA1 and ABCG1 protein expression, thereby increasing Apo-A content. BLEC also enhanced the expression of lipid transport proteins, such as LCAT, and those involved in bile acid metabolism, like CYP7A1.

**Conclusion:**

These results suggest that BLEC's active components may treat HLP by modulating cholesterol metabolism and reducing inflammation and oxidative stress.

## Graphical abstract

**Figure:**
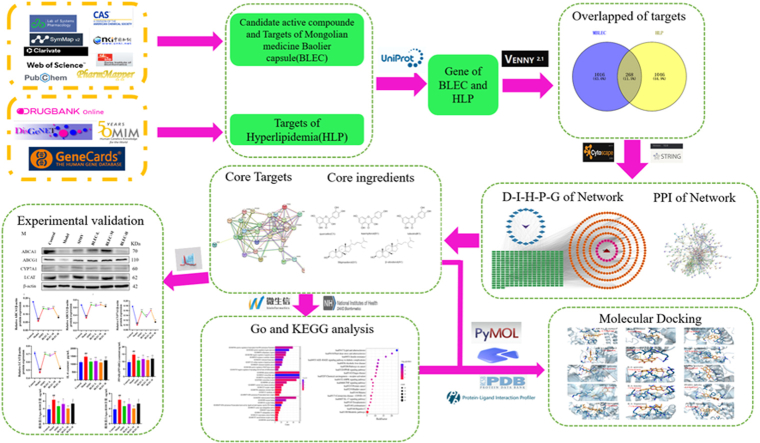

## Abbreviations:

ABCA1: ATP-binding cassette transporter A1
ABCG1: ATP-binding cassette sub-family G member 1
ALT: Alanine aminotransferase
Apo-A1: Apolipoprotein A1
Apo-B100: Apolipoprotein B100
AST: Aspartate aminotransferase
BB: Long Pepper
BLEC: Bao Li Er capsule
CAT: Catalase
CLZ: Chinaberry
CYP7A1: Cytochrome P450 7A1
DH: Rhubarb
D-I-H-P-G: BLEC-Drugs-Ingredients-HLP-Pathway-Gene
DL: Drug like
DS: Salvia miltiorrhiza Bunge
GSH-PX: Glutathione peroxidase
GZ: Ziziphus jujuba
HDL-c: High-density lipoprotein cholesterol
HH: Safflower
HLP: Hyperlipidemia
HQ: Astragalus membranaceus (Fisch.) Bunge
IL-6: Interleukin-6
JX: Rosewood Heart Wood
KZ: Terminalia chebula
LCAT: Lecithin-cholesterol acetyl-transferase
LDL-c: Low-density lipoprotein cholesterol
MD: Dwarf Lilyturf Tuber
MDA: Malondialdehyde
MT: Ophiopogon japonicus
MX: Aucklandiae Radix
NH: Artificial Bezoa
NX: Bovine pericardium
OB: Oral bioavailability
PPAR-γ: Peroxisome proliferator-activated receptor gamma
PPI: Protein-protein Interaction
QC: Rubia cordifolia
RDK: Myristicae semen
SD: Sprague Dawley
SIMV: Simvastatin tablet
SOD: Superoxide dismutase
SQ: Notoginseng
SX: Artificial Moschus
TC: Total cholesterol
TCM: traditional Chinese medicine
TCMSP: Traditional Chinese Medicine Systems Pharmacology Database and Analysis
TG: Triglyceride
TMX: Vladimiria souliei
TX: Sandalwood
ZZ: Gardenia jasminoides

## 1 Introduction

Hyperlipidemia, within the framework of traditional Chinese medicine (TCM), is categorized under the pathologies of phlegm turbidity, lipid turbidity, blood stasis, and damp obstruction. It primarily arises from an accumulation of lipids in the blood, such as cholesterol carried by lipoproteins, triglycerides, phospholipids, and non-esterified fatty acids, due to factors such as unhealthy diet, obesity, and diabetes.^1^^,^^2^ This condition represents one of the most common chronic diseases clinically, characterized by lipid levels exceeding the normal range. Unhealthy dietary habits rich in fats and oils, coupled with psychological and physiological issues, genetics, and diabetes, are key contributors to hyperlipidemia.^3^ Long-term consumption of high-fat diets can lead to lipid metabolism disorders and diseases related to the cardiovascular and immune systems.^4^

In recent years, with the improvement in living standards of families, changes in lifestyle, transportation, and work patterns, along with the global spread of fast-food culture, high-fat diets have rapidly infiltrated people's lives. Additionally, sedentary lifestyles and lack of physical activity have become more prevalent. Factors such as high-altitude, extremely cold climates, and traditional customs have also embedded high-fat diets deeply into the lives of people in both developed and developing countries. In 2020, the prevalence of cardiovascular diseases in the United States remained high, with hyperlipidemia patients aging from 20 to 44 years accounting for 36.1 % of the population.^5^ In China, the prevalence of hyperlipidemia has been increasing annually, with most patients being at high or very high risk, and the overall lipid control rate was only 37.3 % by 2020.^6^ Studies have demonstrated a connection between hyperlipidemia and various metabolic heart diseases, such as obesity, diabetes, hypertension, and coronary heart disease.^7^ Hyperlipidemia is a major contributing factor to arteriosclerotic cardiovascular diseases^8^ and can manifest in forms such as myocardial infarction, cerebral infarction, and various peripheral vascular diseases.^9^

Currently, the primary treatment for hyperlipidemia involves pharmacotherapy, predominantly using statins, fibrates, and niacin, which are associated with adverse reactions like muscle pain, neuropathic pain, muscle enzyme abnormalities, gastrointestinal dysfunctions, and liver and kidney damage.^10^^,^^11^ Statins, in particular, have a reported incidence of muscle pain and weakness ranging from 1 % to 5 % after medication,^12^ necessitating the use of non-statin lipid-lowering drugs. Despite the abundance of research on lowering blood lipid levels, there has been no significant downward trend in the population with hyperlipidemia.^13^ Mongolian medicine, a wisdom crystallized from the Mongolian people's historical experience in dealing with various diseases and mechanisms,^14^ offers new treatment options for hyperlipidemia.

Bao Li Er capsule (BLEC) is formulated based on the understanding of the etiology and pathogenesis of hyperlipidemia within Mongolian medical theory. Drawing upon foundational texts such as *Coral Proven Recipes*, *Collection of Mongolian Medicine Formulas*, and *Supreme Prescriptions*, and guided by traditional Mongolian medical and pharmacological theories, the formula comprises a compound of 21 medicinal ingredients including Ziziphus jujuba, Salvia miltiorrhiza, and Myristica fragrans.^14^ This formulation is designed to enhance cardiac blood supply, promote blood circulation, and thereby facilitate lipid metabolism.^15^ Existing studies on BLEC demonstrate its ability to modulate blood lipid levels in various experimentally-induced hyperlipidemia (HLP) models across multiple species, including rats, mice, and New Zealand rabbits. These studies consistently indicate BLEC's effective regulatory role in HLP. Both acute and chronic toxicity experiments have been conducted, confirming its minimal toxic effects. Furthermore, clinical research, specifically Phase II and III clinical trials, has shown normal readings in routine blood tests and hepatorenal toxicity indicators, with no significant adverse effects or toxic reactions observed. BLEC achieved comparable lipid-lowering efficacy while demonstrating marked improvement in HLP-associated symptoms such as chest tightness, shortness of breath, cardiothoracic pain, dizziness, and fatigue.^16^^,^^17^ Additionally, it effectively ameliorates the progression of atherosclerosis and improves blood lipid profiles, demonstrating therapeutic efficacy in coronary heart disease.^18^ These findings indicate BLEC's promising potential in both novel anti-HLP drug development and clinical applications. However, as a complex formula comprising 21 medicinal ingredients, its preparation process is intricate and cumbersome. Current clinical applications remain relatively limited, and the mechanisms underlying its lipid-lowering effects are not fully elucidated. Moreover, the active pharmaceutical ingredients responsible for treating hyperlipidemia remain unclear, hampering quality control and improvement efforts.

**Fig. 1:**
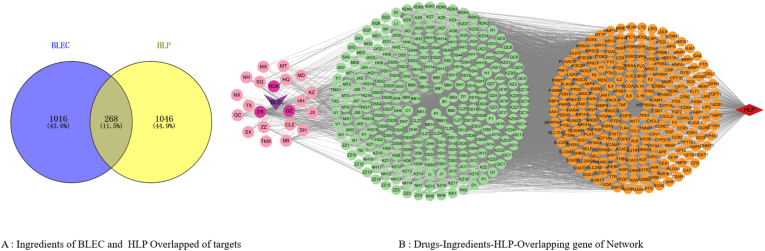
Drug-disease target Venn diagram and drug component-disease-intersection target network diagram. (A) Venn diagram of the intersection targets between drug active components and hyperlipidemia. (B) Network diagram of drug-active component-disease intersection targets.

This study employs network pharmacology methods and molecular docking techniques to predict BLEC's core therapeutic targets and primary pathways in HLP treatment, revealing its active components for lipid reduction (detailed workflow shown in Fig. 1). This research addresses two significant gaps: firstly, it fills the void in understanding the lipid-lowering mechanism and material basis of BLEC' therapeutic effects; secondly, it provides more targeted clinical guidance and options for either independent use in hyperlipidemia treatment or combination therapy for cardiovascular conditions such as hyperlipidemia, lipid-associated hypertension, and hyperglycemia.

## 2 Materials and methods

### 2.1 Materials and reagents

BLEC (Batch No: 2306057) were provided by Inner Mongolia Mongolian Medicine Co., Ltd. (Inner Mongolia, China). Simvastatin tablets (Batch No: X007737) were acquired from Merck Sharp & Dohme (Hangzhou) Pharmaceutical Co., Ltd., (Hangzhou, China). Methanol and acetic acid were purchased from Xilong Scientific (Guangdong, China). Both the rat apolipoprotein A1 (Apo-A1) ELISA kit and the rat apolipoprotein B100 (Apo-B100) ELISA kit were obtained from Wuhan Huamei Biotech Co., Ltd. (Wuhan, China). The Rat peroxisome proliferator activated receptor gamma (PPAR-γ) ELISA kit was purchased from Wuhan EIab Science Co., Ltd. (Wuhan, China), and the rat IL-6 ELISA kit was purchased from Wuhan Boster Biological Technology, Ltd. (Wuhan, China). The anti-ABCA1 antibody and anti-ABCG1 antibody were purchased from Abcam (UK), the CYP7A1 polyclonal antibody from Thermo Fisher Scientific (US), the LCAT antibody from Novus Biologicals (US), and the HRP anti-beta actin antibody from Purdue Bioscience (US). The full-wavelength microplate reader was purchased from Sigma-Aldrich (Shanghai) Trading Co., Ltd. (China). The Bio-Rad Gel Imaging System from Bio-Rich Biotechnology Co., Ltd. (US) was used. All experiments utilized deionized water produced by the Milli-Q system (US).

### 2.2 Network pharmacology

#### 2.2.1 Construction of the active chemical component and target gene database for BLEC

BLEC is a compound formulation consisting of Ziziphus jujuba, Salvia miltiorrhiza, Vladimiria souliei, Rubia cordifolia, Astragalus membranaceus, Gardenia jasminoides, Melia toosendan, Carthamus tinctorius, Ophiopogon japonicus, Panax notoginseng, Terminalia chebula, Dalbergia odorifera, Myristica fragrans, Santalum album, bovine heart, Aucklandia lappa, Rheum palmatum, Akebia quinata, Piper longum, artificial musk, and artificial bezoar. Through searches in databases such as the Traditional Chinese Medicine Systems Pharmacology Database and Analysis Platform (TCMSP, https://www.tcmsp-e.com/tcmsp.php),^19^ SymMap (http://www.symmap.org),^20^ PharmMapper (https://www.lilab-ecust.cn/pharmmapper/), and Web of Science (https://webofscience.clarivate.cn), using the names of the drugs to filter active components. Components were screened based on Oral Bioavailability (OB) ≥30 % and Drug-Likeness (DL) ≥0.18 as primary constituents.^21^^,^^22^ Subsequent retrieval of related active component targets was performed through TCMSP and Swiss Target Prediction (http://www.swisstargetprediction.ch).^23^ These were then imported into the UniProt database (https://www.uniprot.org) for conversion of protein targets to gene targets, organizing the active component-gene target data.^24^

#### 2.2.2 Construction of the HLP target gene database

Disease-related targets were collected through DisGeNET database (https://www.disgenet.org, v7.0), DrugBank (https://go.drugbank.com), OMIM (https://omim.org), and GeneCards (https://www.genecards.org) using the keyword "hyperlipidemia." Additionally, a filtering criterion of a score ≥0.4 was set in GeneCards to ensure relevance and specificity in the collected data.^25^

#### 2.2.3 Construction of the BLEC active Component-HLP target gene database

The target genes of BLEC's active components and the disease target genes were imported into Venny 2.1.0 (https://bioinfogp.cnb.csic.es) for online Venn diagram plotting. This process identified the intersecting targets between BLEC and hyperlipidemia (HLP).

#### 2.2.4 Construction of the BLEC active component-disease target protein-protein interaction (PPI) network

The intersecting genes identified through Venny 2.1.0 (https://bioinfogp.cnb.csic.es) were imported into the STRING database (https://cn.string-db.org/), selecting "Homo sapiens" as the species and setting a confidence threshold of "≥0.9".^26^ The "3D bubble design" option was chosen and disconnected or irrelevant nodes were either hidden or removed to obtain the protein-protein interaction network diagram and data file for the intersecting genes between BLEC and hyperlipidemia. The PPI network diagram of BLEC active components, hyperlipidemia, and target genes was then constructed using Cytoscape 3.10.1 software.

#### 2.2.5 GO gene function annotation analysis and KEGG enrichment analysis

The intersecting genes between components and disease targets were imported into the DAVID database (https://david.ncifcrf.gov), selecting "Homo sapiens" as the species. The predicted core targets underwent Gene Ontology (GO) analysis and Kyoto Encyclopedia of Genes and Genomes (KEGG) pathway enrichment analysis. This included analysis of biological processes, cellular components, molecular functions, and signaling pathways, with the results presented in bar graphs. Relevant data tables were compiled and packaged as files.

### 2.3 Animal experiments

#### 2.3.1 Ethical statement and modeling

All procedures in this experiment were conducted in accordance with the recommendations for the use and care of animals outlined by the National Institutes of Health (NIH) in the United States. The animal experiment protocol was approved by the Laboratory Animal Management and Ethics Committee of Jiangxi University of Chinese Medicine on July 26th, 2023, with the ethical approval number: JZLLSC20230076. Furthermore, all animal experiments were carried out in accordance with the relevant content of the ARRIVE guidelines.

Sixty male Sprague Dawley (SD) rats of SPF grade, weighing 170 ± 10 g, were purchased from Hunan SJA Laboratory Animal Co., Ltd., with a production license number SCXK (Xiang) 2019-0004. The experiment was conducted at the Experimental Animal Science and Technology Center of Jiangxi University of Chinese Medicine, with an animal use permit number: SYXK (Gan) 2022-0002. The high-fat diet used for modeling primarily consisted of basic feed, 15 % lard, 20 % sucrose, 5 % casein, 1.2 % cholesterol, and 0.2 % sodium cholate, which was purchased from Xiaomi Youtai (Beijing) Biotechnology Co., Ltd., with a license number SCXK (Jing) 2018-0006. Daily food intake was recorded, and all rats were weighed after a week to establish their initial weight.

#### 2.3.2 Administration method, dosage, and duration

Phase one: High-fat diet modeling. After a week of acclimatization, the rats were weighed on the 8th day to record their initial body weight. They were randomly divided into two groups: a blank group of 10 rats fed with maintenance feed and the remaining 50 rats subjected to a high-fat, high-cholesterol diet containing bile salts,^27^ with free access to water and maintained under the same environmental conditions. After 4 weeks of modeling, the experiment proceeded to phase two.

Phase two: Drug treatment. After successful modeling, the rats were randomly divided into five groups of 10 each. The normal group (referred to as "Control") was given basic feed and an equivalent volume of saline 10 mL/kg daily; the model control group (referred to as "Model") was given high-fat feed and an equivalent volume of saline 10 mL/kg daily; the Simvastatin group (referred to as "SIMV") was given high-fat feed and dosed daily with a Simvastatin solution at 2 mg/kg; the BLEC low, medium, and high dose groups (referred to as "BLEC-L″, "BLEC-M″, "BLEC-H″, respectively) were fed a high-fat diet, with the medium dose calculated based on the normal human dosage adjusted for rat body surface area, amounting to 0.46 g/kg. The low and high doses were half and twice the medium dose, respectively. The medication was administered by gavage once a day for 8 weeks, during which the rats' diet, weight, and other physical signs were monitored and recorded.

To examine dose-dependency, the medium dose (BLEC-M) was calculated based on the standard adult human dosage of this traditional Chinese medicine compound, converted according to the body surface area of rats. The low dose (BLEC-L) was set at 0.5 times the medium dose, while the high dose (BLEC-H) was set at 2 times the medium dose. This dosing strategy aligned with the dosage selection in previous research conducted by Hao Gang et al.^16^

#### 2.3.3 Sample collection and biomarker measuring

##### 2.3.3.1 Behavioral observations

During the modeling and medication phases, observations were made regarding the rats' food intake (increased, decreased, average), fur condition (clean and shiny, slightly yellow and dull, coarse, disordered, bristled, etc.), mental state (active, changes in activity level, lethargic and sleepy, lackluster), and feces (quantity, color, moisture content, consistency). These observations were recorded.

##### 2.3.3.2 Tissue collection and sample processing

On the last day of administration, after a 12-h fasting period with water allowed, the rats were weighed and then anesthetized. Blood was collected from the abdominal aorta, and after euthanasia, the liver was photographed and weighed. The right outer lobe of the liver was excised and fully immersed in 4 % paraformaldehyde solution for fixation, in preparation for making liver tissue pathological sections. The left outer lobe was reserved for liver indicator measuring, and the triangular lobe was used for Western blot analysis. Blood samples were left to stand at room temperature for 0.5–1 h, then centrifuged at 4 °C at 3500–4500 rpm in a high-speed refrigerated centrifuge, for 10–15 min per cycle, to collect the supernatant for further analysis.

##### 2.3.3.3 Liver index changes and HE staining

The entire liver was carefully dissected from the animal, rinsed with saline, dried, and weighed. The liver index was calculated using the formula: liver weight/body weight × 100 %, taking into account the rats' weight recorded previously.

Liver tissues fixed in 4 % paraformaldehyde were taken out, rinsed, and sections approximately 2 cm × 1.5 cm in size and no more than 3 mm thick were prepared. These sections were placed in embedding frames and subjected to gradient dehydration in varying concentrations of ethanol, cleared in xylene, and embedded in paraffin for 3 h. The sections were then sliced, baked, and mounted for staining with hematoxylin and eosin. Finally, the slides were sealed, and liver tissue pathological changes were observed and photographed under a light microscope.

##### 2.3.3.4 Serum and liver tissue biomarker measuring

Serum samples were taken and processed in strict accordance with the instructions provided in the reagent kits. Blood samples were collected from the abdominal aorta of anesthetized rats. The samples were centrifuged at 3500–4500 r/min for 10–15 min at 4 °C using a refrigerated high-speed centrifuge. The supernatant was collected to obtain serum for subsequent analysis. The contents of total cholesterol (TC), triglycerides (TG), low-density lipoprotein cholesterol (LDL-c), and high-density lipoprotein cholesterol (HDL-c) in the serum, as well as the enzyme activities of aspartate aminotransferase (AST) and alanine aminotransferase (ALT), were measured. Concurrently, liver tissues were rinsed with ice-cold saline to remove any residual blood, dried with filter paper, and 100 ± 10 mg of liver tissue was weighed and placed into centrifuge tubes with 3 steel balls. Following the ratio of liver tissue (g) to saline (mL) = 1:9, saline was added, and the tissues were homogenized using a tissue homogenizer. After centrifugation at low temperature and high speed, the supernatant obtained was used as a 10 % liver tissue homogenate. The activities of malondialdehyde (MDA), superoxide dismutase (SOD), glutathione peroxidase (GSH-Px), and catalase (CAT) were then determined according to the instructions of the test kits. Data were analyzed using one-way ANOVA with GraphPad Prism 9.3.0 and SPSS 27 software.

##### 2.3.3.5 Western blotting for protein expression analysis

For the liver tissues, 50 mg was placed into centrifuge tubes, and 2 % SDS lysis buffer was added at a ratio of tissue (mg) to 2 % SDS (mL) = 50:1. The tissues were ground using a tissue grinder, and proteins were extracted by centrifugation, then denatured in a 100 °C metal bath for 15 min. Protein concentration was determined according to the BCA protein assay kit instructions. A 40 μL protein sample was mixed with 5 × loading buffer and separated by SDS-PAGE gel electrophoresis, followed by wet transfer to a membrane, and blocked with 5 % skim milk. The sample was incubated overnight at 4 °C with antibodies against ABCA1, ABCG1, CYP7A1, LCAT, and β-actin. The next day, after incubation with secondary antibodies at room temperature, the membrane was exposed and developed using the horseradish peroxidase-ECL method. Protein band images and grayscale value data were collected using Image Lab software. Antibody dilution ratios are as shown in Table 1, and data analysis was performed using GraphPad Prism 9.3.0.

**Table 1:** Dilution ratio of each antibody.

Antibody name	Source	Dilution Ratio
Anti-ABCA1 antibody	Abcam	1:2500
Anti-ABCG1 antibody	Abcam	1:1000
CYP7A1 Polyclonal antibody	Thermo-Fisher	1:500
LCAT antibody	Novus Biologicals	1:500
HRP Anti-Beta Actin antibody	Purdue Bioscience	1:5000

##### 2.3.3.6 Data analysis

Statistical evaluation and analysis of data from all groups were conducted using SPSS software (version 27). Subsequently, one-way analysis of variance (ANOVA) was performed on the data using GraphPad Prism to generate the analysis graphs.

### 2.4 Molecular docking validation

Utilizing the Traditional Chinese Medicine Systems Pharmacology Database and Analysis Platform (TCMSP), the molecular structures of the core active components were obtained. Target protein PDB IDs were retrieved through literature review, and the target protein structure files were acquired from the Protein Data Bank website (https://www.rcsb.org/).^28^ The AutoDock 1.1.2 software was employed for dehydration and dehydrogenation of proteins and small molecules, leading to the formation of ligands and receptors. The PyMOL 2.5.5.7 software facilitated the visualization of the binding sites between the relevant targets of hyperlipidemia (HLP) and the core components of BLEC. Additionally, the bound compounds were obtained using Discovery Studio 2021, ChemDraw 20.0, and the Protein-Ligand Interaction Profiler (PLIP) web page (https://plip-tool.biotec.tu-dresden.de/).^29^ The post-docking 2D structural diagrams were drawn to illustrate the interactions.

## 3 Results

### 3.1 Network pharmacology

#### 3.1.1 Construction of the BLEC active chemical components and target gene database

Through the TCMSP, SymMap, PharmMapper, and Web of Science databases, a total of 318 active chemical components were identified (as listed in Table 1s in supplementary material), including 9 from Ziziphus jujuba (GZ), 65 from Salvia miltiorrhiza (DS), 9 from Myristica fragrans (RDK), 1 from Vladimiria souliei (TMX), 19 from Rubia cordifolia (QC), 20 from Astragalus membranaceus (HQ), 15 from Gardenia jasminoides (ZZ), 9 from Melia toosendan (CLZ), 22 from Carthamus tinctorius (HH), 5 from Ophiopogon japonicus (MD), 8 from Panax notoginseng (SQ), 37 from Terminalia chebula (KZ), 37 from Dalbergia odorifera (JX), 3 from Santalum album (TX), 2 from bovine heart (NX), 6 from Aucklandia lappa (MX), 16 from Rheum palmatum (DH), 8 from Akebia quinata (MT), 15 from Piper longum (BB), 7 from artificial musk (SX), and 5 from artificial bezoar (NH). A total of 13,727 active component targets were retrieved, and after converting targets using the UniProt database, merging duplicate target genes, and removing targets without corresponding genes, 1284 target genes were identified, forming the target gene database for the active components of BLEC.

#### 3.1.2 Construction of the hyperlipidemia disease target gene database

**Fig. 2:**
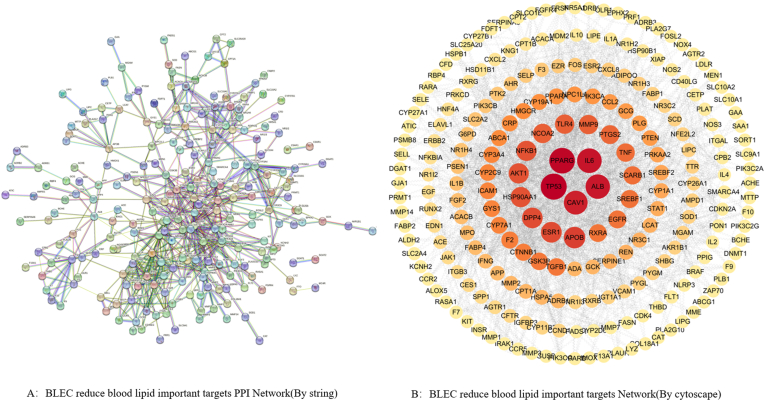
Active component-disease intersection target PPI network protein interaction diagram. (A) PPI diagram of the intersection targets between drug active components and disease. (B) Network diagram of the intersection targets between drug active components and disease.

By searching the DisGeNET, DrugBank, OMIM, and GeneCards databases with the keyword "hyperlipidemia" and setting a filtering criterion of score ≥0.4 in GeneCards,^25^ a total of 1314 disease target genes were compiled after merging targets from all four databases and removing duplicates. Specifically, 19 targets were obtained from DisGeNET, 32 from DrugBank, 53 from OMIM, and 1676 from GeneCards, as shown in Fig. 2s.

#### 3.1.3 Construction of the BLEC active components-hyperlipidemia target gene database

The target genes of the active chemical components from the 21 ingredients of BLEC and the disease target genes were imported into Venny 2.1.0 for online Venn diagram plotting. This process identified 268 intersecting targets between BLEC and hyperlipidemia, which may represent important targets for the lipid-lowering effects of BLEC. The Venn diagram of intersecting targets is shown in Fig. 1A. Using Cytoscape 3.10.1 software, a "Drugs-Ingredients-HLP-Overlapping gene" network diagram was constructed (Fig. 1B).

#### 3.1.4 Construction of the BLEC active components-disease targets and key targets protein-protein interaction (PPI) network

**Fig. 3:**
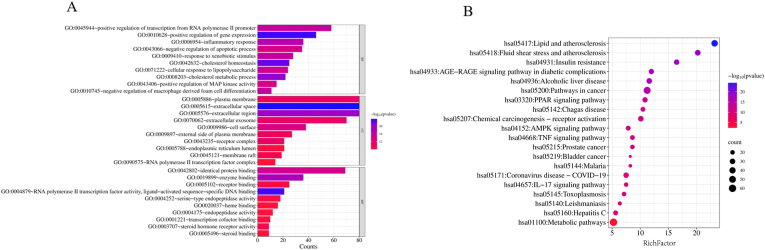
Results of GO (A) and KEGG (B) enrichment analysis for BLEC treatment of HLP. Fig. 3(A) shows the top 10 Gene Ontology (GO) enrichment analysis results for BLEC treatment of HLP. Fig. 3(B) presents the top 20 Kyoto Encyclopedia of Genes and Genomes (KEGG) analysis results for BLEC treatment of HLP.

The intersecting target genes of BLEC active components and hyperlipidemia, obtained through Venny 2.1.0, were analyzed on the STRING platform. The analysis yielded a network with 268 nodes, 620 edges, an average node degree of 4.63, and an average local clustering coefficient of 0.473. The intersecting gene PPI network diagram was exported (Fig. 2A). The analysis results were then imported into Cytoscape 3.10.1 software. Utilizing the CytoNCA plugin and based on degree, betweenness, and closeness values, the top 62 target genes were sorted and intersected, identifying 28 key targets. The topological parameters are presented in Table 2, and a network diagram of "BLEC active components-hyperlipidemia intersecting target genes" was constructed (Fig. 2B). The darker the color and the larger the bubble, the higher the density of protein interactions. Additionally, a PPI network diagram of the 28 key targets was obtained (Fig. 3s).

**Table 2:** Topological parameters of 28 core target proteins in BLEC treatment for hyperlipidemia.

No.	Core target	Betweenness	Closeness	Degree
1	TP53	6818.2026	0.34681183	56
2	PPARG	6598.5293	0.33037037	28
3	IL6	6068.34	0.34254992	50
4	ALB	5941.495	0.2969374	22
5	CAV1	5614.542	0.3378788	26
6	ESR1	4008.7737	0.34681183	38
7	HSP90AA1	3751.17	0.33993903	42
8	AKT1	3729.2231	0.3441358	48
9	NFKB1	3715.5022	0.3430769	36
10	NCOA2	3309.152	0.30094466	28
11	TLR4	3281.0852	0.3383915	36
12	MMP9	3273.331	0.30464482	26
13	PTGS2	3014.5435	0.31721196	18
14	TNF	2773.604	0.33383232	46
15	EGFR	2521.8572	0.3462733	40
16	RXRA	2439.2659	0.28299493	30
17	TGFB1	2361.5552	0.32365748	20
18	GSK3B	2311.1123	0.30298913	22
19	CTNNB1	2124.891	0.3383915	34
20	ICAM1	1862.5918	0.30886427	28
21	ABCA1	1609.1525	0.27736318	14
22	PIK3CA	1412.8539	0.31948423	34
23	CCL2	1346.0385	0.30053908	30
24	PTEN	1312.4768	0.30929264	30
25	STAT1	1027.3711	0.3208633	22
26	IFNG	814.52075	0.30674002	36
27	FGF2	741.69684	0.28480205	14
28	IL1B	734.4022	0.31857142	40

**Fig. 4:**
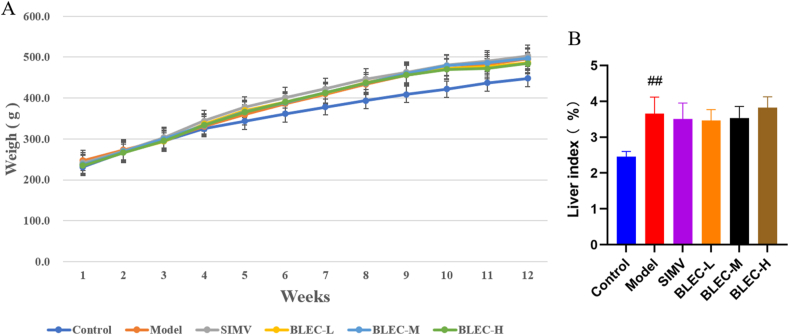
Changes in body weight cycles and one-way ANOVA analysis of liver index (%) bar graph for different groups of rats. (A) is the line graph of body weight changes from modeling to drug administration over 12 weeks for the different groups of rats. (B) shows the results of the one-way ANOVA analysis of the liver index for six groups of rats (^##^*P* < 0.01 vs. control group).

Similarly, based on the "Drugs-active components-intersecting targets" gene database obtained from Venny 2.1.0 and imported into Cytoscape software, and by sorting the top 15 based on degree, betweenness, and closeness values and taking their intersection, 5 key active components were identified: quercetin, beta-sitosterol, kaempferol, luteolin, and stigmasterol, as shown in Table 3 and supplementary material 2 (Fig. 4s). These five components may play a significant role in the lipid-lowering effect of BLEC.

**Table 3:** BLEC's 5 core chemical components for reducing blood lipids.

MOL-ID	Abbr.	Ingredient	Degree	OB%	DL	Drug
MOL000098	C1	Quercetin	573	46.43	0.28	Choerospondias axillaries, Astragalus membranaceus (Fisch.) Bunge, Gardenia, Cape Jasmine, Chinaberry, Safflower, Notoginseng
MOL000358	A1	Beta-sitosterol	243	36.91	0.75	Choerospondias axillaries, Rubia cordifolia, Gardenia, Cape Jasmine, Safflower, Notoginseng, Rosewood Heart Wood, Myristicae Semen, Rhubarb, Ophiopogon japonicus
MOL000422	B1	Kaempferol	195	41.88	0.24	Choerospondias axillaries, Astragalus membranaceus (Fisch.) Bunge, Gardenia, Cape Jasmine, Safflower
MOL000006	K1	Luteolin	179	36.16	0.25	Salvia miltiorrhiza Bunge, Safflower, Sandalwood (TX), Terminalia chebula,
MOL000449	S1	Stigmasterol	130	43.83	0.76	Gardenia, Cape Jasmine, Safflower, Notoginseng, Aucklandiae Radix, Ophiopogon japonicus

#### 3.1.5 Gene Ontology (GO) functional annotation analysis

The 268 intersecting genes between BLEC components and disease were imported into the DAVID database for functional annotation analysis. Relevant biological processes, cellular components, and molecular functions were selected based on a *p*-value <0.05. A total of 612 BPs, 76 CCs, and 131 MFs were identified. The top 10 terms based on Counts values were selected for the construction of an enrichment bubble chart (Fig. 3A). The main biological processes in which BLEC are involved include positive regulation of gene expression, cholesterol homeostasis, cholesterol metabolic process, inflammatory response, response to xenobiotic stimulus, and positive regulation of transcription from RNA polymerase II promoter. The primary cellular components involved are the extracellular space, extracellular region, cell surface, extracellular exosome, receptor complex, and plasma membrane. The significant molecular functions include identical protein binding, enzyme binding, receptor binding, RNA polymerase II transcription factor activity with ligand-activated sequence-specific DNA binding, serine-type endopeptidase activity, and heme binding.

#### 3.1.6 Kyoto encyclopedia of genes and genomes (KEGG) pathway analysis

**Fig. 5:**
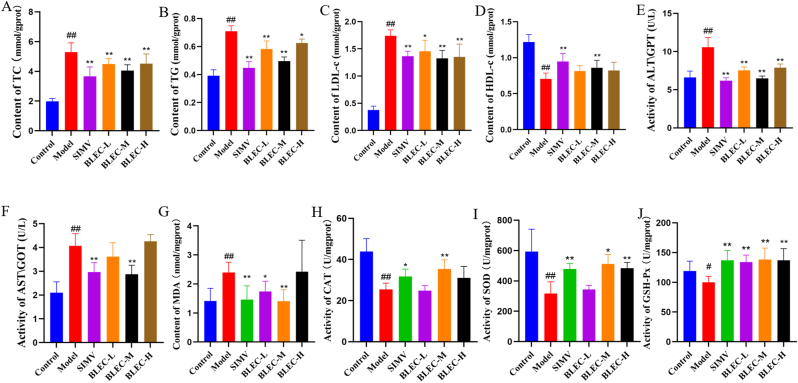
Bar Graphs of Biochemical Indices in Different Groups of Rats Based on One-way ANOVA Analysis. (A) shows the results of the one-way ANOVA analysis of TC in different groups treated with BLEC for HLP. (B) shows the results of the one-way ANOVA analysis of TG in different groups treated with BLEC for HLP. (C) shows the results of the one-way ANOVA analysis of LDL-c in different groups treated with BLEC for HLP. (D) shows the results of the one-way ANOVA analysis of HDL-c in different groups treated with BLEC for HLP. (E) shows the results of the one-way ANOVA analysis of ALT in different groups treated with BLEC for HLP. (F) shows the results of the one-way ANOVA analysis of aspartate AST in different groups treated with BLEC for HLP. (G) shows the results of the one-way ANOVA analysis of MDA in different groups treated with BLEC for HLP. (H) shows the results of the one-way ANOVA analysis of CAT in different groups treated with BLEC for HLP. (I) shows the results of the one-way ANOVA analysis of SOD in different groups treated with BLEC for HLP. (J) shows the results of the one-way ANOVA analysis of GSH-px in different groups treated with BLEC for HLP (^##^*P* < 0.01 vs. control group, ∗∗*P* < 0.01, ∗*P* < 0.05 vs. model group).

Using the DAVID platform for KEGG enrichment analysis of the intersecting genes, similar to the previous analysis, based on a *p*-value <0.05, 146 enriched signaling pathways were identified. The top 20 pathways were selected for bubble chart visualization, as shown in Fig. 3B. The main related signaling pathways include the AGE-RAGE signaling pathway in diabetic complications, PPAR signaling pathway, AMPK signaling pathway, TNF signaling pathway, among others. Notably, the PPAR receptor is involved in physiological processes such as lipid metabolism, cell proliferation, and differentiation, closely related to fatty acid oxidation and metabolism. The bluer the bubble, the more significant and higher the enrichment of the pathway^30^; the larger the bubble, the greater the number of enriched genes. The results (Fig. 3B) facilitated the construction of a "Drug-Component-Disease-Pathway-Gene (BLEC-Drugs-Ingredients-HLP-Pathway-Gene, abbreviated as D-I-H-P-G)" network using Cytoscape software, further elucidating the relationships between components, targets, and pathways. As shown in Fig. 5s, a network of 259 targets, 318 active components, and 20 KEGG pathways was established. Through topological analysis with the software, the five compounds with the highest degree values—quercetin, beta-sitosterol, kaempferol, luteolin, and stigmasterol—were identified as the core active components of BLEC, aligning with the results of the previous screening.

### 3.2 Validation by animal experiment

#### 3.2.1 Establishment of the hyperlipidemia model

##### 3.2.1.1 Behavioral changes

Compared to the control group, the model group rats exhibited a decrease in food intake, their fur gradually became dull and slightly yellow, they were mostly lethargic and slept more, and their movement decreased. Their feces were mostly brownish-yellow, moist, and soft.

##### 3.2.1.2 Body weight, liver index, and appearance

**Fig. 6:**
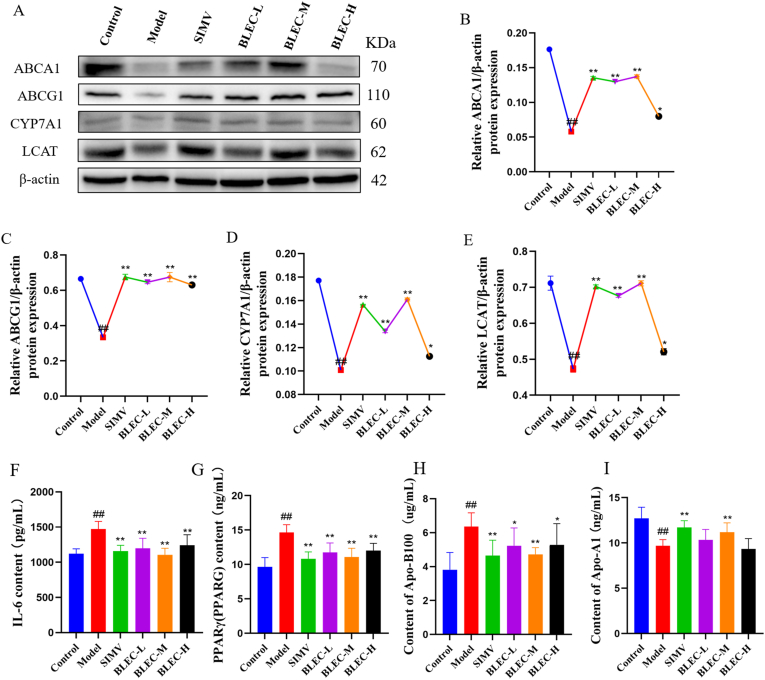
Western blot band density values and ELASA kit detection data for different groups of rats with one-way ANOVA analysis results. Fig. 6(A) shows the bands obtained from Western blot experiments for the four protein antibodies ABCA1, ABCG1, CYP7A1, and LCAT, along with the internal reference β-actin. Fig. 6(B) presents the one-way ANOVA analysis results of the ratio of band density values for antibody ABCA1 across different groups to the internal reference β-actin. Fig. 6(C) presents the one-way ANOVA analysis results of the ratio of band density values for antibody ABCG1 across different groups to the internal reference β-actin. Fig. 6(D) presents the one-way ANOVA analysis results of the ratio of band density values for antibody CYP7A1 across different groups to the internal reference β-actin. Fig. 6(E) presents the one-way ANOVA analysis results of the ratio of band density values for antibody LCAT across different groups to the internal reference β-actin. Fig. 6(F) presents the one-way ANOVA analysis results of the IL-6 detection data obtained through the ELASA kit. Fig. 6(G) presents the one-way ANOVA analysis results of the PPARγ detection data obtained through the ELASA kit. Fig. 6(H) presents the one-way ANOVA analysis results of the Apo-A1 detection data obtained through the ELASA kit. Fig. 6(I) presents the one-way ANOVA analysis results of the Apo-B100 detection data obtained through the ELASA kit. (^##^*P* < 0.01 vs. control group, ∗∗*P* < 0.01, ∗*P* < 0.05 vs. model group).

There was no significant difference in body weight growth between the model group and the control group (Fig. 4A). However, a noticeable increase in the liver index was observed in the model group (Fig. 4B). Examination of the liver appearance (Fig. 6s) revealed that compared to the control group, the livers in the model group were longer, enlarged, and thickened, with a pale color and dense lipid droplets, clearly visualizing the differences between the two groups.

##### 3.2.1.3 Lipid index measurement

Compared to the control group (Table 4), the serum levels of TC and TG in the model group were significantly elevated (P < 0.001), indicating that the high-fat diet significantly increased the rats' lipid levels. This clearly demonstrated the success of the model establishment, thereby allowing the experiment to proceed to the next phase.

**Table 4:** Comparison of blood lipids after four weeks of modeling (x ± s).

Group	Sample count	Dose	TC/mmol/L	TG/mmol/L
Control	10	–	1.45 ± 0.03	0.82 ± 0.25
Model	50	–	3.00 ± 0.33^##^	1.05 ± 0.30

Note: ^##^ indicates *P* < 0.01 vs. control group.

#### 3.2.2 The regulatory effect of BLEC on HLP

Data analysis results (Fig. 5) showed that compared to the control group, the model group rats showed a significant increase in serum TC, TG, and LDL levels (*P* < 0.01), and a significant decrease in HDL levels (Fig. 5A, B, 5C, 5D, *P* < 0.01). The activities of AST and ALT enzymes were also significantly increased (Fig. 5E and F, *P* < 0.01), indicating that the high-fat diet successfully established a hyperlipidemia model and caused liver damage in rats. In comparison with the model group, after 8 weeks of medication, the treatment group showed trends in the opposite direction for the aforementioned indicators (Fig. 5D, *P* < 0.05), suggesting that BLEC can indeed regulate liver lipid and cholesterol metabolism. This finding is consistent with a previous report.^31^

Additionally, as shown in the results (Fig. 5), compared to the control group (Fig. 5G, H, 5I, 5J), the model group rats exhibited a significant decrease in the activities of SOD, GSH-PX and CAT enzymes (*P* < 0.05), and a significant increase in MDA content (*P* < 0.05). This preliminarily reflected that a long-term high-fat diet can cause severe oxidative stress and inflammatory responses in rats, leading to significant organismal damage and increased lipo-toxicity.^32^ Compared to the model group, the BLEC-M group rats showed a significant increase in the activities of SOD, GSH-PX, and CAT enzymes (*P* < 0.05), and a significant decrease in MDA content (*P* < 0.05). Although the BLEC-L and BLEC-H groups did not show significant changes for some indicators (*P* > 0.05), there was a trend towards improvement. This suggests that BLEC can also regulate liver lipid metabolism function by enhancing liver antioxidative capacity and reducing tissue damage, with the medium dose showing the best effect.

Based on the above results, it was evident that BLEC is effective in reducing and alleviating the elevated blood lipids, liver damage, and oxidative stress caused by a high-fat diet, thereby treating hyperlipidemia.

#### 3.2.3 The impact of BLEC on liver tissue in HLP

**Fig. 7:**
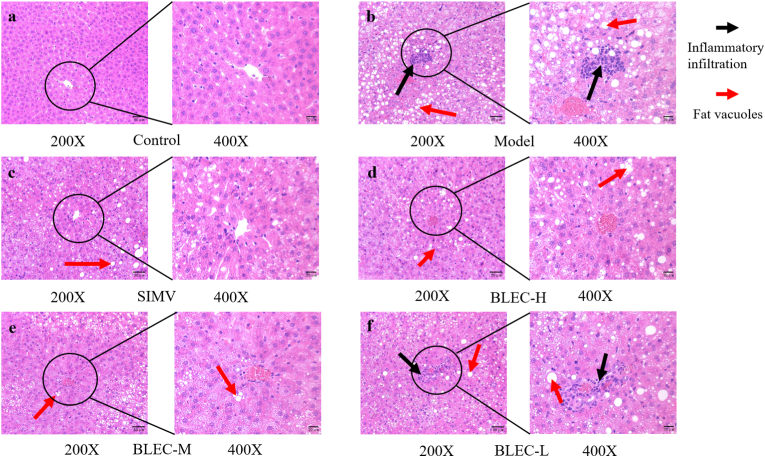
Photomicrographs of liver pathology sections at 200 to 400x magnification from 6 groups of rats treated with BLEC for HLP (a, Normal group; b, Model group; c, Simvastatin group; d, High-dose BLEC; e, Medium-dose BLEC; f, Low-dose BLEC).

As shown in Fig. 7, compared to the control group, liver cells in the model group were significantly swollen, with disorganized cell arrangement, large areas of lipid droplet accumulation, and evident inflammatory cell infiltration. In comparison with the model group, liver cells in the SIMV group showed slight swelling, with a more organized arrangement, very small areas of fat vacuoles, and no inflammatory cell infiltration. The BLEC-H group exhibited swollen liver cells, relatively organized arrangement, medium areas of fat vacuole accumulation, and no signs of inflammatory cell infiltration. The liver tissue morphology of the BLEC-M group was closer to normal, with an organized cell arrangement, very few fat vacuoles, and virtually no inflammatory cell infiltration. In the BLEC-L group, some liver cells were still swollen, with a more disordered arrangement, medium amounts of fat vacuole accumulation, and signs of inflammatory cell infiltration. All these findings inferred that BLEC has a certain alleviating effect on the inflammatory response, liver pathology, and lipid accumulation in the liver caused by a high-fat diet, with the medium dose showing the best effect.

#### 3.2.4 The effect of active components in BLEC on the expression of core targets

To explore the material basis and mechanism of BLEC in treating hyperlipidemia, this study employed Western blotting and ELISA kits to verify the predictive results of network pharmacology. Additionally, literature review revealed that ABCA1 and ABCG1 are important membrane proteins involved in the transport of lipid molecules within the body. They play a major role in promoting the binding of free cholesterol and phospholipids to the liver cell receptor Apo-A1 and the formation of HDL-c.^33^^,^^34^ On the other hand, Apo-B100, the carrier protein of LDL, primarily stimulates the generation and esterification of cholesterol in vascular smooth muscle cells, thereby accelerating lipid deposition.^35^ CYP7A1 is the rate-limiting enzyme in bile acid synthesis and plays a crucial role in lipid metabolism within the liver.^36^ Furthermore, Lecithin-Cholesterol Acyltransferase (LCAT) is key in the transport of free TC to the liver for breakdown and metabolism. It promotes the esterification of cholesterol in plasma, facilitating cholesterol reverse transport, mainly acting on HDL protein; it also plays a vital role in catalyzing the esterification reaction of cholesterol in plasma.^37^^,^^38^

Combining the results from network pharmacology GO and KEGG analyses, the experiment ultimately selected ABCA1, ABCG1, LCAT, and CYP7A1 for detection using Western blotting techniques. Additionally, ELISA kits were used to measure the levels of carrier proteins Apo-A1 and Apo-B100, as well as IL-6 and PPARG (PPARγ). The results (Fig. 6F-I) showed that compared to the control group, the high-fat diet-induced hyperlipidemia significantly downregulated the expression of ABCA1 and ABCG1 proteins (*P* < 0.01) and also significantly downregulated LCAT and CYP7A1 proteins (*P* < 0.01) in rats. Compared to the model group, the treatment group showed a significant upregulation in the expression of ABCA1, ABCG1, LCAT, and CYP7A1 proteins (*P* < 0.05).

Furthermore, ELISA kit determination results (Fig. 6F-I) indicated that compared to the control group, the levels of IL-6, PPARG, and Apo-B100 were significantly increased (*P* < 0.01), while the level of carrier protein Apo-A1 was significantly decreased (*P* < 0.01). Compared to the model group, the MBIEC group significantly reduced the levels of IL-6, PPARG, and Apo-B100 (*P* < 0.05) and significantly increased the level of Apo-A1 (*P* < 0.05). These results demonstrate that the dose-response relationship of BLEC in treating HLP is BLEC-M > BLEC-L > BLEC-H.

In summary, BLEC may exert its therapeutic effects on HLP by modulating the expression of proteins such as ABCA1, ABCG1, LCAT, and CYP7A1, and by regulating the levels of carrier proteins Apo-A1, Apo-B100, and the contents of IL-6 and PPARG. This regulation promotes cholesterol transport, bile acid metabolism, and reverses inflammatory responses, thereby achieving the goal of treating HLP.

### 3.3 Molecular docking results

Based on the predictions from the PPI and D-I-H-P-G networks, the study ultimately selected five core active components of BLEC for their lipid-lowering effects: quercetin, beta-sitosterol, kaempferol, luteolin, and stigmasterol. These components were docked with three key target proteins directly related to the lipid-lowering potential mechanisms of BLEC, identified through topological analysis among the top 28 targets: IL-6 (PDB ID: 1ALU),^39^ PPARG (PDB ID: 1PRG),^40^ and ABCA1 (PDB ID: 5XJY).^41^ The results (as shown in Fig. 7s) indicated that the binding energies between each target protein's pocket and the five active components were all less than −5 kcal/mol. This suggested that the core chemical components of BLEC have good affinity and stability with the main targets of HLP, consistent with literature reports that these compounds have beneficial effects against HLP.^42^, ^43^, ^44^, ^45^, ^46^ This further confirmed that BLEC exerts its therapeutic effects on HLP through multi-target interactions.

In conclusion, combining the results of animal experiments, quercetin, beta-sitosterol, kaempferol, luteolin, and stigmasterol in BLEC may exert their therapeutic effects on HLP by modulating the expression of ABCA1, ABCG1, LCAT, and CYP7A1 proteins, as well as regulating the levels of carrier proteins Apo-A1, Apo-B100, IL-6, and PPARG. This regulation facilitates cholesterol transport, bile acid metabolism, and reverses inflammatory and oxidative stress responses, thereby achieving the goal of treating HLP.

## 4 Discussion

### 4.1 Key active components and pathways in BLEC's treatment of hyperlipidemia

Based on the predictions from the PPI and D-I-H-P-G networks constructed through network pharmacology, combined with literature research, it has been discovered that active components in BLEC, such as quercetin, beta-sitosterol, kaempferol, luteolin, and stigmasterol, play a significant role within these networks. In the PPI network, BLEC significantly interacts with key HLP-related targets including IL-6, PPARG, and ABCA1. The PPARG protein is closely associated with obesity and hypercholesterolemia,^47^ while ABCA1 plays a crucial role in lipid metabolism.^48^

Through Gene Ontology (GO) and Kyoto Encyclopedia of Genes and Genomes (KEGG) enrichment analyses, 819 significant (P < 0.05) GO processes and 146 KEGG pathways were identified. The molecular function enrichment results primarily involved processes such as the positive regulation of gene expression, cholesterol homeostasis, cholesterol metabolic process, and inflammatory response. This led to the speculation that cholesterol metabolism and inflammatory response are the main biological processes through which BLEC treats HLP. The KEGG pathway enrichment results indicated that BLEC mainly treats HLP by participating in pathways such as the AGE-RAGE signaling pathway in diabetic complications, the PPAR signaling pathway, the AMPK signaling pathway, and the TNF signaling pathway. These pathways are highly congruent with cholesterol metabolism and inflammatory response, likely representing the main mechanisms through which BLEC treats HLP. Therefore, the results of this experiment suggested that modulating cholesterol metabolism and combating inflammatory responses are the primary therapeutic mechanisms of BLEC in treating hyperlipidemia.

### 4.2 The role of BLEC's active components in lipid metabolism and liver health

Quercetin, an active component in BLEC, plays a pivotal role in lipid metabolism by inhibiting the levels of TC, TG and LDL-c. This leads to a reduction in lipid deposition caused by high-fat diets, improvement in liver damage and fatty degeneration, and antioxidative effects, thereby enhancing liver function and modulating lipid metabolism mechanisms.^49^^,^^50^ Beta-sitosterol, a natural sterol, contributes to lowering lipid levels through its anti-inflammatory and antioxidative actions, as well as promoting the expression of genes involved in lipid metabolism. Experimental research indicated that kaempferol could decrease the accumulation of TG in the liver, thus facilitating lipid metabolism in liver cells.^51^ luteolin is involved in regulating enzymes that modulate liver lipid metabolism, thereby reducing the content of fatty acids and cholesterol in liver cells.^52^ Stigmasterol, a natural plant sterol, lowers LDL-c level by inhibiting the intestinal absorption of cholesterol. It mediates the expression of ABCG1 protein through the PPAR signaling pathway, promoting the binding of cholesterol to carrier proteins and its reverse transport into the liver. This also stimulates the expression of CYP7A1 protein, enhancing the conversion of cholesterol into bile acids and thus regulating bile acid metabolism for a lipid-lowering effect.^53^ Quercetin, kaempferol, and luteolin, which are all flavonoids, exert a regulatory effect on the PPAR signaling pathway.^54^

Furthermore, in this experiment, a hyperlipidemia model was established using SD rats treated with BLEC. The results showed that BLEC significantly reduced the levels of TC, TG and LDL-c, decreased the activity of AST and ALT enzymes, and significantly increased the level of HDL-c. This demonstrated that BLEC not only has a potent lipid-lowering effect but also regulates liver lipid metabolism by alleviating liver damage.

### 4.3 BLEC's mechanism in treating hyperlipidemia: inflammation and oxidative stress modulation

Through literature review and experimental validation, it has been discovered that BLEC treats hyperlipidemia by inhibiting inflammatory and oxidative stress responses. BLEC can suppress the levels of IL-6, a key inflammatory factor in cellular inflammatory stress responses, reducing the body's adaptive resistance to inflammation and thereby exacerbating inflammatory damage. The AGE-RAGE signaling pathway plays a central role in this process.^55^ Additionally, BLEC significantly reduced the body's MDA, a product of lipid peroxidation, indicating its protective role against superoxide free radical damage and oxidative stress. This was achieved through the activation of SOD, an important antioxidant enzyme.^56^ BLEC also enhanced the activity of GSH-PX and CAT, contributing to the protection of cellular membrane structure and function.^57^ Furthermore, BLEC exhibited a mitigating effect on the liver pathological changes caused by HLP.^58^^,^^59^

On the other hand, BLEC also exerted lipid-lowering effects by inhibiting the levels of PPARG. PPAR receptors include PPAR-α, PPAR-β, and PPAR-γ (that is, PPARG), which are involved in physiological processes such as lipid metabolism, cell proliferation and differentiation, and are closely related to the oxidation and metabolism of fatty acids.^30^ Furthermore, it mediated the expression of ABCA1, ABCG1, CYP7A1, and LCAT through the PPAR signaling pathway to regulate lipid level in the bloodstream, increasing the content of the carrier protein Apo-A1 and reducing the level of the lipoprotein Apo-B100, thereby exerting a regulatory function in lipid metabolism. ABCA1, ABCG1, and CYP7A1 were identified as significant target proteins within the cholesterol metabolic process and the PPAR signaling pathway.^60^ ABCA1 and ABCG1, in particular, are important membrane proteins involved in the transport of lipid molecules within the body. Together, they play a pivotal role in facilitating the binding of free cholesterol and phospholipids to the liver cell receptor APO-A1 and the formation of HDL-c, thereby promoting the reverse transport of cholesterol to the liver and regulating hepatic cholesterol metabolism.^33^^,^^34^ Apo-B100, acting as the carrier protein for LDL, primarily stimulates the production and esterification of cholesterol in vascular smooth muscle cells, thus accelerating the deposition of lipids.^35^^,^^61^ CYP7A1, a rate-limiting enzyme in the synthesis of bile acids, plays a crucial role in the metabolism of lipids within the liver.^36^^,^^62^ Concurrently, LCAT acts as a key enzyme in the transport of free TC to the liver for breakdown and metabolism, promoting the esterification of cholesterol in plasma and facilitating the reverse transport of cholesterol. It plays a significant role in carrying HDL proteins and is also crucial in catalyzing the esterification reaction of cholesterol in plasma.^37^^,^^38^

### 4.4 Comprehensive analysis of BLEC's mechanism in treating hyperlipidemia

This study utilized network pharmacology to predict the five core components of BLEC, as well as the potential targets and main pathways through which BLEC treats hyperlipidemia. Based on these predictions, we identified several important biological processes and pathways potentially related to the treatment of HLP with BLEC, providing a basis for further network pharmacology experiments. The experiment selected IL-6, PPARG and ABCA1 as the core targets for the treatment of HLP with BLEC. Molecular docking results also showed that the five key active components could easily enter and stably bind to the active pockets of the target proteins, interacting with key amino acids at the binding sites.

Furthermore, animal experimental results confirmed that compared to the control group, the model group of rats showed a significant increase in IL-6 and PPARG levels and a significant downregulation of ABCA1 protein expression. Conversely, compared to the model group, IL-6 and PPARG levels significantly decreased, and ABCA1 protein expression significantly increased in the BLEC-treated group. Therefore, we believe that BLEC treats HLP by regulating cholesterol metabolism and combating inflammatory and oxidative stress responses. The consistency between molecular docking, animal experimental validation, and network pharmacology predictions underscores the accuracy of this method in deducing the pharmacodynamic material basis and mechanism of action of BLEC.

This experiment, while exploring the material basis and mechanisms of BLEC in treating hyperlipidemia, has also revealed several issues warranting further investigation. When comparing doses, the medium dose (equivalent to the normal adult dosage) of BLEC showed superior efficacy. Although the results for low and high doses in regulating lipid metabolism, anti-inflammatory, and antioxidative effects, as well as protein expression, were not significantly notable, they all exhibited a trend towards alleviating HLP. Consequently, we hypothesize that the relationship between the dose of BLEC and its therapeutic effect follows a "parabolic" trend. In this scenario, BLEC-M, positioned around the peak of the parabola, can be considered the optimal drug concentration, while the low and high doses are on either side. The therapeutic efficacy is not as high or decreases when the dosage is below or above this optimal concentration. This also corroborates the correctness of the dosage settings in the standards for BLEC (2010 edition).

### 4.5 Benefits/risks versus statins

Advantages: According to current clinical medication reports and practical observations in hyperlipidemia treatment, compared with statin lipid-lowering drugs, BLEC have demonstrated no significant acute or chronic toxicity in preclinical studies. Furthermore, routine blood tests and other indicators remained normal during and after Phase II and III clinical trials, with no significant toxic side effects or adverse reactions observed, while achieving equivalent lipid-lowering efficacy. Additionally, BLEC exhibit beneficial effects in reducing blood viscosity, prolonging coagulation time and normobaric hypoxia tolerance, inhibiting platelet aggregation, decreasing thrombus wet weight, effectively reducing fibrinogen levels, and providing sedative and analgesic effects. These properties make it particularly effective in treating complex cardiovascular diseases, such as coronary heart disease with dyslipidemia, and provide an option for combination therapy in cases where monotherapy shows insufficient efficacy or requires more targeted treatment approaches.

Disadvantages: Comparing to Western statin medications, BLEC, as a complex Traditional Mongolian medicine formula comprising 21 medicinal ingredients, presents complex and cumbersome formulation and preparation processes, with some ingredients being difficult to obtain. Moreover, as a Category III Mongolian medicine, BLEC are primarily used in combination therapy, with relatively few clinical trials examining its use as monotherapy. Current clinical trials have been predominantly conducted in Mongolian regions, where lifestyle habits, climate conditions, and physical constitutions differ significantly from other regions. The lack of trials in other geographical areas has resulted in regional differences in drug administration and population adaptability, potentially leading to limitations in applicability.

### 4.6 Limitation and clinical implications

Firstly, as the results were derived from network pharmacology database screening, such predictions are inherently limited and cannot be considered the exclusive pathway through which BLEC exerts their lipid-lowering effects. Secondly, the dose-independent response observed during in vivo validation experiments suggests potential toxicity reactions, though this hypothesis requires further investigation and verification in future studies.

This study elucidates the lipid-lowering mechanism of BLEC and its material basis for therapeutic efficacy, providing valuable reference for clinical application and quality control. Furthermore, it offers mechanistic guidance and therapeutic options for its use, either as monotherapy for hyperlipidemia or in combination therapy for complex cardiovascular conditions, including hyperlipidemia, hypertension with hyperlipidemia, hyperglycemia, and other combined cardiovascular disorders. This enhanced understanding enables more targeted clinical medication strategies.

## 5 Conclusion

This study, through network pharmacology analysis, molecular docking, and in vivo experimental validation, investigated the lipid-lowering effects, pharmacodynamic material basis, and molecular mechanisms of the Mongolian medicine BLEC. Based on network pharmacology, we identified quercetin, beta-sitosterol, kaempferol, luteolin, and stigmasterol as the five core active components of BLEC. Through the PPI network, IL-6, PPARG, and ABCA1 were selected as core targets among 28 key targets, offering new insights into the treatment of hyperlipidemia. Gene Ontology and Kyoto Encyclopedia of Genes and Genomes enrichment analyses suggested that regulating cholesterol metabolism and combating inflammatory responses might be crucial processes and pathways in BLEC's treatment of HLP. Moreover, molecular docking results demonstrated good stability and affinity between the active components and core targets. Animal experimental results further indicated that the treatment of HLP with BLEC is associated with a reduction in IL-6 and PPARG levels and an upregulation of ABCA1 protein expression. In summary, the experiment provides an effective approach to understanding the pharmacodynamic material basis and the mechanism of BLEC treating HLP, which is through regulating cholesterol metabolism and combating inflammatory responses. This study offers reliable experimental evidence for further clinical research and application of BLEC.

## CRediT authorship contribution statement

**Xuelan Fu:** Investigation, data collection and analysis, Writing – review & editing. **Jiehong Xing:** Funding acquisition, Resources. **Chengjun Yuan:** Methodology, adviser. **Qingling Liu:** Funding acquisition, Resources. **Jixiao Zhu:** Methodology. **Jinxiang Zeng:** Methodology. **Haisha Lu:** data collection. **Huiqing Li:** data collection. **Guoyue Zhong:** Resources. **Jian Liang:** experiment design, Supervision, Writing – review & editing.

## Ethical statement

All animal procedures strictly adhered to the regulations set by the Laboratory Animal Management and Ethics Committee of Jiangxi University of Chinese Medicine, which was approved with number JZLLSC20230076 on July 26th, 2023.

## Declaration of competing interest

The authors declare the following financial interests/personal relationships which may be considered as potential competing interests: Jian Liang reports financial support was provided by Inner Mongolia Mongolian Medicine Co., Ltd. Jian Liang reports financial support was provided by Inner Mongolia Mongolian Medicine Co., Ltd. If there are other authors, they declare that they have no known competing financial interests or personal relationships that could have appeared to influence the work reported in this paper.
