# Supplementary material for: A comprehensive study on the underlying mechanisms of the lipid-lowering effects of Bao Li Er Capsule in hyperlipidemia
Source: J Tradit Complement Med. 2025 Mar 21;16(1):96–108. doi: 10.1016/j.jtcme.2025.03.006 (PMC12902230; doi:10.1016/j.jtcme.2025.03.006)
Supplement: Multimedia component 1 [file mmc1.docx]

Figure 1s Workflow diagram for BLEC treatment of HLP based on network pharmacology, molecular docking and animal experimental analysis.
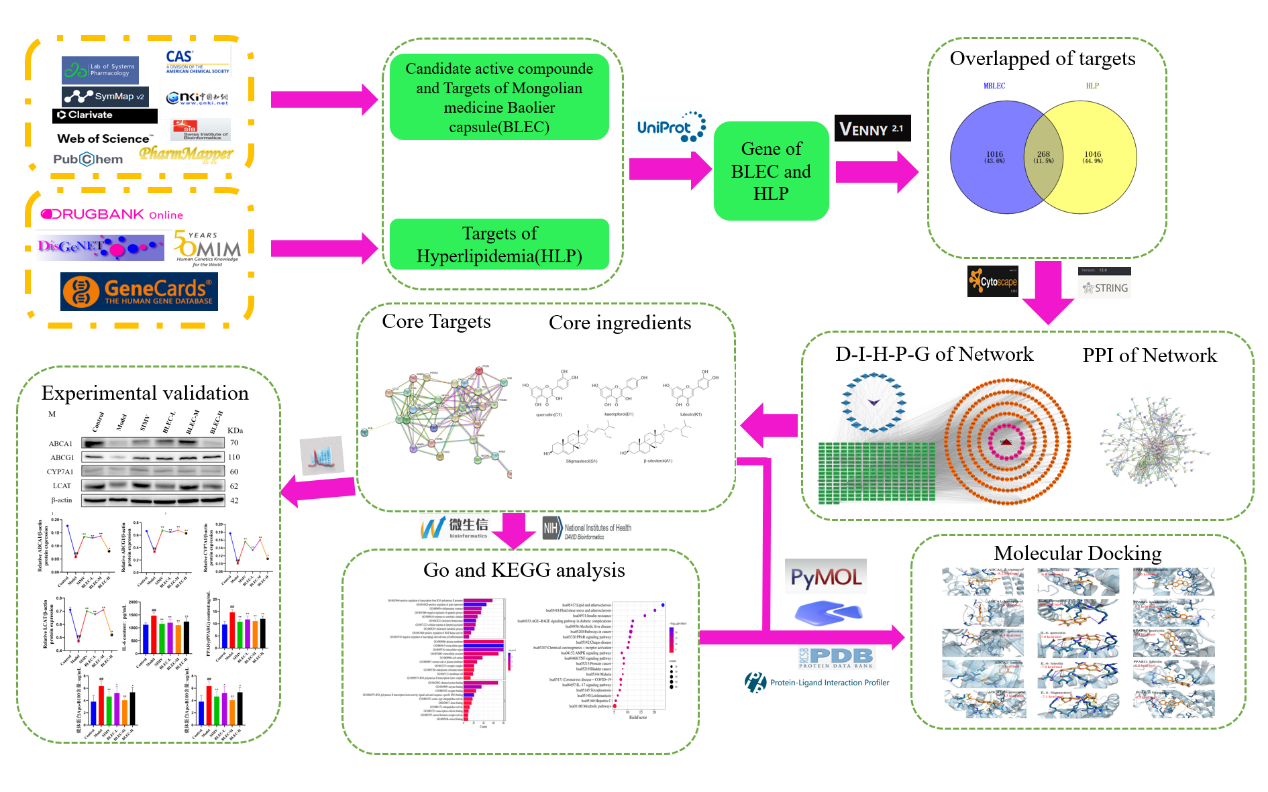


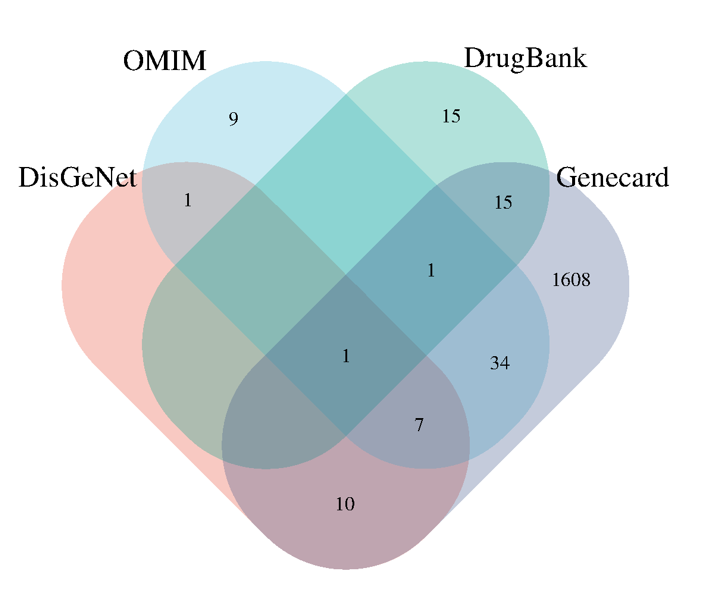
Figure 2s Venn diagram of hyperlipidemia target retrieval from 4 databases.


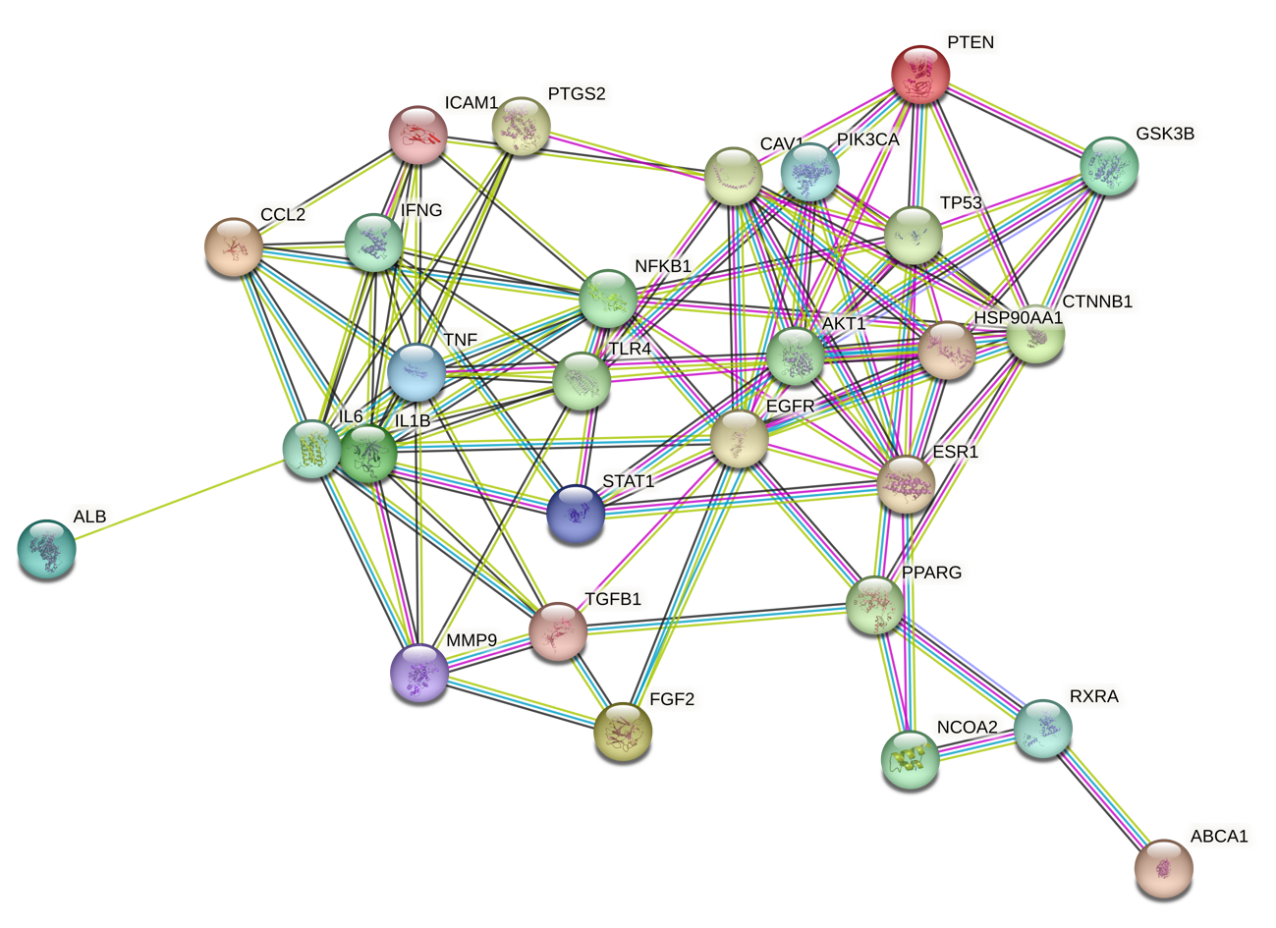
Figure 3 PPI network diagram of 28 core targets.


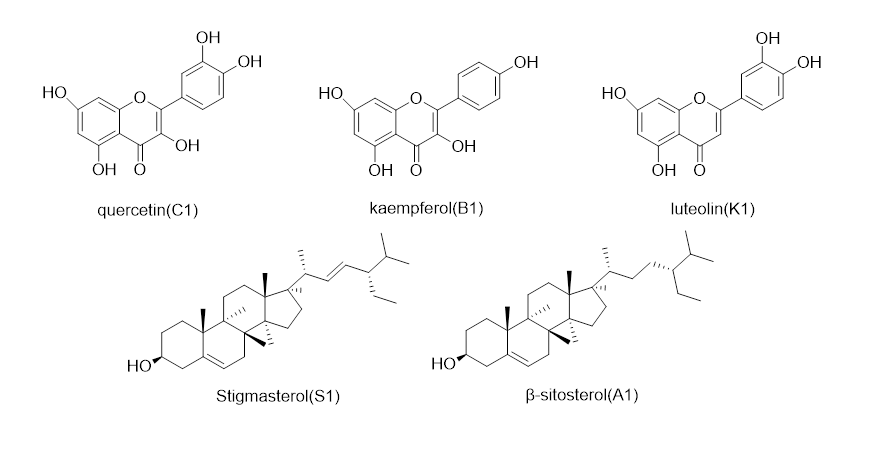
Figure 4s Chemical structure of the 5 core components of BLEC


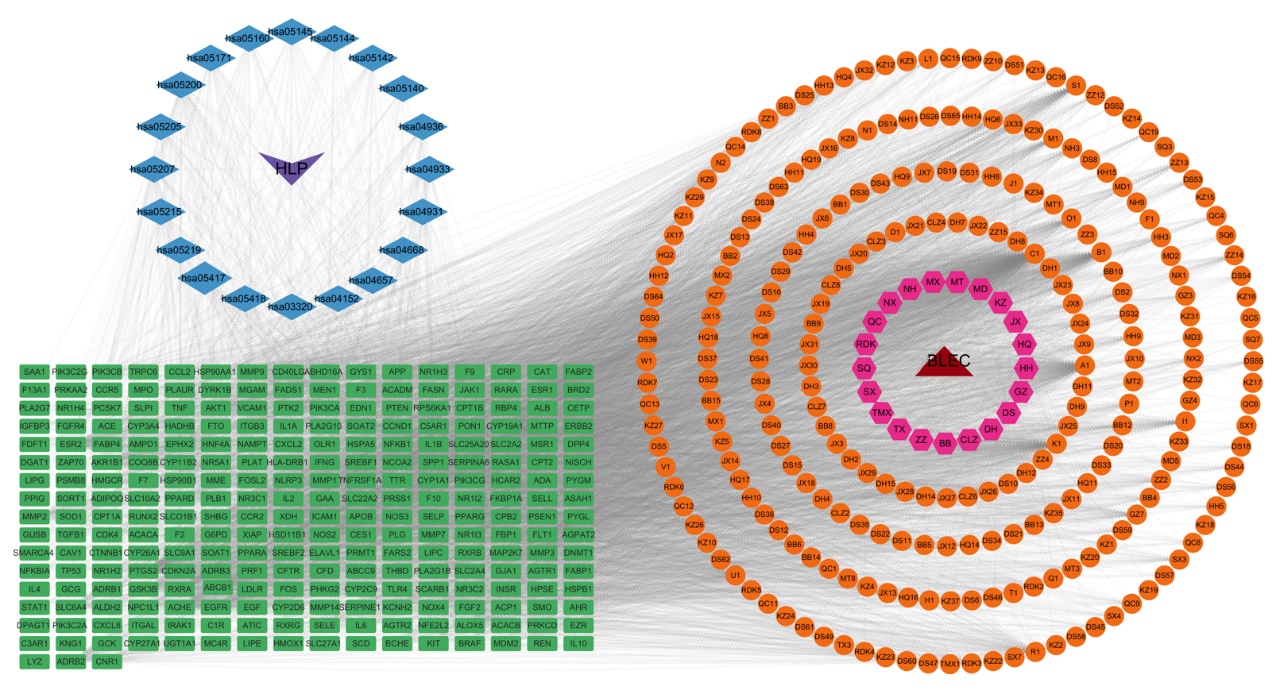
Figure 5s BLEC-Drugs-Ingredients-HLP-Pathway-Gene (D-I-H-P-G) PPI Network Diagram.


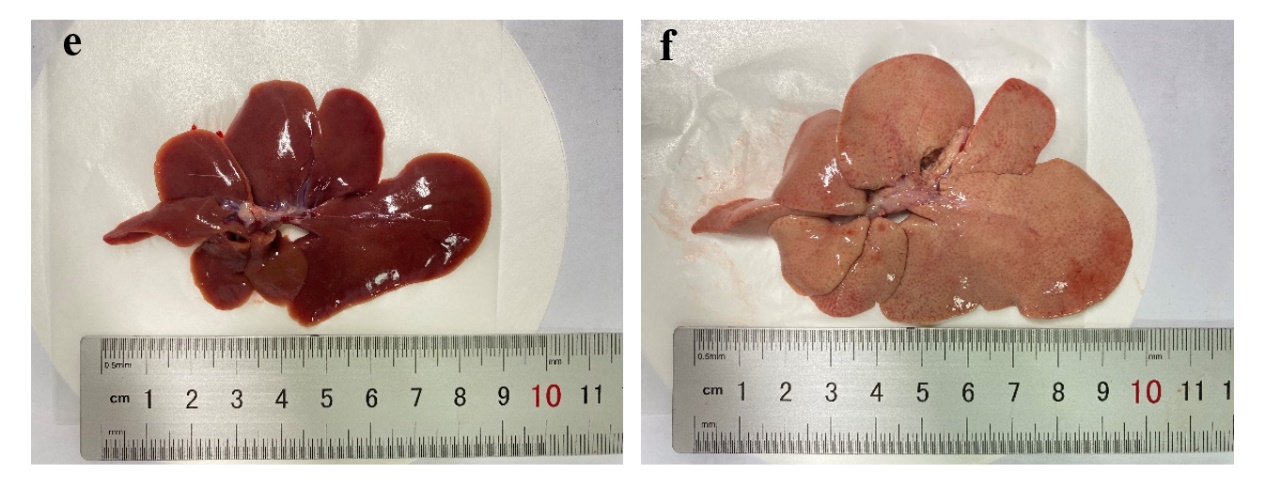
Figure 6s Liver appearance of rats in the control group (e) and model group (f).


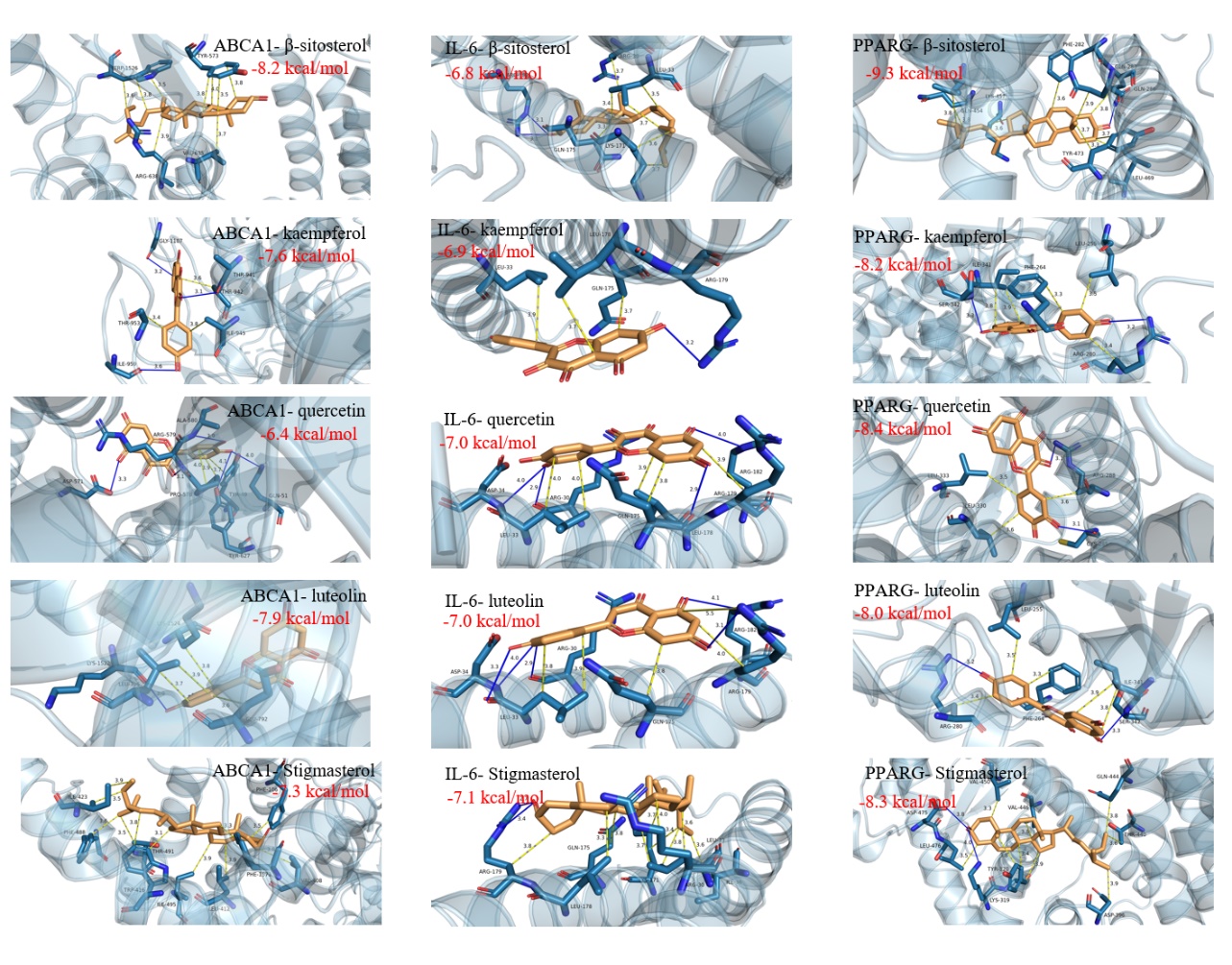
Figure 7s Molecular docking results of IL-6, PPARG, and ABCA1 proteins with BLEC's five core active components (quercetin, beta-sitosterol, kaempferol, luteolin and stigmasterol).

Table 1s Active components in 21 ingredient medications of BLEC

| Drug name | Abbreviations | Mol ID | Molecule Name | OB (%) | DL |
| --- | --- | --- | --- | --- | --- |
| Choerospondias axillaries  (GZ) | W1 | MOL001002 | Ellagic acid | 43.06 | 0.43 |
|  | D1 | MOL001040 | (2R)-5,7-dihydroxy-2-(4-hydroxyphenyl)chroman-4-one | 42.36 | 0.21 |
|  | GZ3 | MOL001490 | bis[(2S)-2-ethylhexyl] benzene-1,2-dicarboxylate | 43.59 | 0.35 |
|  | GZ4 | MOL001736 | (-)-Taxifolin | 60.51 | 0.27 |
|  | A1 | MOL000358 | Beta-sitosterol | 36.91 | 0.75 |
|  | B1 | MOL000422 | Kaempferol | 41.88 | 0.24 |
|  | GZ7 | MOL004328 | Naringenin | 59.29 | 0.21 |
|  | F1 | MOL000096 | (-)-Catechin | 49.68 | 0.24 |
|  | C1 | MOL000098 | Quercetin | 46.43 | 0.28 |
| Salvia miltiorrhiza Bunge  (DS) | L1 | MOL001601 | 1,2,5,6-tetrahydrotanshinone | 38.75 | 0.36 |
|  | DS2 | MOL001659 | Poriferasterol | 43.83 | 0.76 |
|  | I1 | MOL001771 | Poriferast-5-en-3beta-ol | 36.91 | 0.75 |
|  | H1 | MOL001942 | Isoimperatorin | 45.46 | 0.23 |
|  | DS5 | MOL002222 | Sugiol | 36.11 | 0.28 |
|  | DS6 | MOL002651 | Dehydrotanshinone II A | 43.76 | 0.4 |
|  | J1 | MOL002776 | Baicalin | 40.12 | 0.75 |
|  | DS8 | MOL000569 | Digallate | 61.85 | 0.26 |
|  | K1 | MOL000006 | Luteolin | 36.16 | 0.25 |
|  | DS10 | MOL006824 | α-Amyrin | 39.51 | 0.76 |
|  | DS11 | MOL007036 | 5,6-dihydroxy-7-isopropyl-1,1-dimethyl-2,3-dihydrophenanthren-4-one | 33.77 | 0.29 |
|  | DS12 | MOL007041 | 2-isopropyl-8-methylphenanthrene-3,4-dione | 40.86 | 0.23 |
|  | DS13 | MOL007045 | 3α-hydroxytanshinoneⅡa | 44.93 | 0.44 |
|  | DS14 | MOL007048 | (E)-3-[2-(3,4-dihydroxyphenyl)-7-hydroxy-benzofuran-4-yl]acrylic acid | 48.24 | 0.31 |
|  | DS15 | MOL007049 | 4-methylenemiltirone | 34.35 | 0.23 |
|  | DS16 | MOL007050 | 2-(4-hydroxy-3-methoxyphenyl)-5-(3-hydroxypropyl)-7-methoxy-3-benzofurancarboxaldehyde | 62.78 | 0.4 |
|  | DS17 | MOL007051 | 6-o-syringyl-8-o-acetyl shanzhiside methyl ester | 46.69 | 0.71 |
|  | DS18 | MOL007058 | Formyltanshinone | 73.44 | 0.42 |
|  | DS19 | MOL007059 | 3-beta-Hydroxymethyllenetanshiquinone | 32.16 | 0.41 |
|  | DS20 | MOL007061 | Methylenetanshinquinone | 37.07 | 0.36 |
|  | DS21 | MOL007063 | Przewalskin a | 37.11 | 0.65 |
|  | DS22 | MOL007064 | Przewalskin b | 110.32 | 0.44 |
|  | DS23 | MOL007068 | Przewaquinone B | 62.24 | 0.41 |
|  | DS24 | MOL007069 | Przewaquinone c | 55.74 | 0.4 |
|  | DS25 | MOL007070 | (6S,7R)-6,7-dihydroxy-1,6-dimethyl-8,9-dihydro-7H-naphtho[8,7-g]benzofuran-10,11-dione | 41.31 | 0.45 |
|  | DS26 | MOL007071 | Przewaquinone f | 40.31 | 0.46 |
|  | DS27 | MOL007077 | Sclareol | 43.67 | 0.21 |
|  | DS28 | MOL007079 | Tanshinaldehyde | 52.47 | 0.45 |
|  | DS29 | MOL007081 | Danshenol B | 57.95 | 0.56 |
|  | DS30 | MOL007082 | Danshenol A | 56.97 | 0.52 |
|  | DS31 | MOL007085 | Salvilenone | 30.38 | 0.38 |
|  | DS32 | MOL007088 | Cryptotanshinone | 52.34 | 0.4 |
|  | DS33 | MOL007093 | Dan-shexinkum d | 38.88 | 0.55 |
|  | DS34 | MOL007094 | Danshenspiroketallactone | 50.43 | 0.31 |
|  | DS35 | MOL007098 | Deoxyneocryptotanshinone | 49.4 | 0.29 |
|  | DS36 | MOL007100 | Dihydrotanshinlactone | 38.68 | 0.32 |
|  | DS37 | MOL007101 | DihydrotanshinoneⅠ | 45.04 | 0.36 |
|  | DS38 | MOL007105 | Epidanshenspiroketallactone | 68.27 | 0.31 |
|  | DS39 | MOL007107 | C09092 | 36.07 | 0.25 |
|  | DS40 | MOL007108 | Isocryptotanshi-none | 54.98 | 0.39 |
|  | DS41 | MOL007111 | Isotanshinone II | 49.92 | 0.4 |
|  | DS42 | MOL007115 | Manool | 45.04 | 0.2 |
|  | DS43 | MOL007118 | Microstegiol | 39.61 | 0.28 |
|  | DS44 | MOL007119 | Miltionone Ⅰ | 49.68 | 0.32 |
|  | DS45 | MOL007120 | Miltionone Ⅱ | 71.03 | 0.44 |
|  | DS46 | MOL007121 | Miltipolone | 36.56 | 0.37 |
|  | DS47 | MOL007122 | Miltirone | 38.76 | 0.25 |
|  | DS48 | MOL007123 | Miltirone Ⅱ | 44.95 | 0.24 |
|  | DS49 | MOL007124 | Neocryptotanshinone ii | 39.46 | 0.23 |
|  | DS50 | MOL007125 | Neocryptotanshinone | 52.49 | 0.32 |
|  | DS51 | MOL007127 | 1-methyl-8,9-dihydro-7H-naphtho[5,6-g]benzofuran-6,10,11-trione | 34.72 | 0.37 |
|  | DS52 | MOL007130 | Prolithospermic acid | 64.37 | 0.31 |
|  | DS53 | MOL007132 | (2R)-3-(3,4-dihydroxyphenyl)-2-[(Z)-3-(3,4-dihydroxyphenyl)acryloyl]oxy-propionic acid | 109.38 | 0.35 |
|  | DS54 | MOL007140 | (Z)-3-[2-[(E)-2-(3,4-dihydroxyphenyl)vinyl]-3,4-dihydroxy-phenyl]acrylic acid | 88.54 | 0.26 |
|  | DS55 | MOL007141 | Salvianolic acid g | 45.56 | 0.61 |
|  | DS56 | MOL007142 | Salvianolic acid j | 43.38 | 0.72 |
|  | DS57 | MOL007143 | Salvilenone Ⅰ | 32.43 | 0.23 |
|  | DS58 | MOL007145 | Salviolone | 31.72 | 0.24 |
|  | DS59 | MOL007149 | NSC 122421 | 34.49 | 0.28 |
|  | DS60 | MOL007150 | (6S)-6-hydroxy-1-methyl-6-methylol-8,9-dihydro-7H-naphtho[8,7-g]benzofuran-10,11-quinone | 75.39 | 0.46 |
|  | DS61 | MOL007151 | Tanshindiol B | 42.67 | 0.45 |
|  | DS62 | MOL007152 | Przewaquinone E | 42.85 | 0.45 |
|  | DS63 | MOL007154 | Tanshinone iia | 49.89 | 0.4 |
|  | DS64 | MOL007155 | (6S)-6-(hydroxymethyl)-1,6-dimethyl-8,9-dihydro-7H-naphtho[8,7-g]benzofuran-10,11-dione | 65.26 | 0.45 |
|  | DS65 | MOL007156 | Tanshinone Ⅵ | 45.64 | 0.3 |
| Myristicae Semen (RDK) | A1 | MOL000358 | Beta-sitosterol | 36.91 | 0.75 |
|  | RDK2 | MOL007920 | Meso-1,4-bis-(4-hydroxy-3-methoxyphenyl)-2,3-dimethylbutane | 31.32 | 0.26 |
|  | RDK3 | MOL009243 | Isoguaiacin | 48.78 | 0.31 |
|  | RDK4 | MOL009254 | Galbacin | 61 | 0.53 |
|  | RDK5 | MOL009255 | 5-[(2S,3S)-7-methoxy-3-methyl-5-[(E)-prop-1-enyl]-2,3-dihydrobenzofuran-2-yl]-1,3-benzodioxole | 53.11 | 0.4 |
|  | RDK6 | MOL009259 | Kudos | 45.06 | 0.38 |
|  | RDK7 | MOL009263 | Saucernetindiol | 41.85 | 0.32 |
|  | RDK8 | MOL009264 | Tetrahydrofuroguaiacin B | 62.86 | 0.32 |
|  | RDK9 | MOL009265 | Threo-austrobailignan-5 | 49.49 | 0.32 |
| Inulae radix (TMX) | TMX1 | MOL010836 | Isoalantolactone | 53.4306 |  |
| Rubia cordifolia (QC) | QC1 | MOL003283 | (2R,3R,4S)-4-(4-hydroxy-3-methoxy-phenyl)-7-methoxy-2,3-dimethylol-tetralin-6-ol | 66.51 | 0.39 |
|  | A1 | MOL000358 | Beta-sitosterol | 36.91 | 0.75 |
|  | M1 | MOL000359 | Sitosterol | 36.91 | 0.75 |
|  | QC4 | MOL005638 | Mollugin | 42.34 | 0.26 |
|  | QC5 | MOL005869 | Daucostero_qt | 36.91 | 0.75 |
|  | QC6 | MOL006139 | 1,3-dimethoxy-2-carboxyanthraquinone | 102.89 | 0.33 |
|  | QC7 | MOL006141 | 1,3-dihydroxy-2-hydroxymthylanthraquinone-3-O-xylosyl(1→6)-glucoside_qt | 71.27 | 0.27 |
|  | QC8 | MOL006147 | Alizarin-2-methylether | 32.81 | 0.21 |
|  | QC9 | MOL006149 | 7-hydroxy-8-methyl-4-vinyl-9,10-dihydrophenanthrene-1-carboxylic acid | 56.99 | 0.27 |
|  | QC10 | MOL006150 | 1-acetoxy-6-hydroxy2-methylanthraquinone-3-O-α-rhamnosyl(1→4)-α-glucoside | 30.74 | 0.64 |
|  | QC11 | MOL006153 | 2'-hydroxymollugin | 40.5 | 0.29 |
|  | QC12 | MOL006155 | 4-hydroxy-9,10-dioxoanthracene-2-Carboxylic acid | 45.98 | 0.25 |
|  | QC13 | MOL006160 | Alizarin | 32.67 | 0.19 |
|  | QC14 | MOL006162 | Nordamnacanthal | 53.97 | 0.24 |
|  | QC15 | MOL006164 | Pallasone | 43.87 | 0.4 |
|  | QC16 | MOL006167 | Methyl 6-hydroxy-2,2-dimethyl-3,4-dihydrobenzo[h]chromene-5-carboxylate | 51.09 | 0.25 |
|  | QC17 | MOL006170 | Henine | 77.12 | 0.24 |
|  | QC18 | MOL006171 | Rubiprasin B | 35.97 | 0.68 |
|  | QC19 | MOL006174 | Xyloidone | 31.61 | 0.18 |
| Astragalus membranaceus (Fisch.) Bunge (HQ) | P1 | MOL000211 | Mairin | 55.38 | 0.78 |
|  | HQ2 | MOL000239 | Jaranol | 50.83 | 0.29 |
|  | Q1 | MOL000296 | Hederagenin | 36.91 | 0.75 |
|  | HQ4 | MOL000033 | (3S,8S,9S,10R,13R,14S,17R)-10,13-dimethyl-17-[(2R,5S)-5-propan-2-yloctan-2-yl]-2,3,4,7,8,9,11,12,14,15,16,17-dodecahydro-1H-cyclopenta[a]phenanthren-3-ol | 36.23 | 0.78 |
|  | O1 | MOL000354 | Isorhamnetin | 49.6 | 0.31 |
|  | HQ6 | MOL000371 | 3,9-di-O-methylnissolin | 53.74 | 0.48 |
|  | HQ7 | MOL000374 | 5'-hydroxyiso-muronulatol-2',5'-di-O-glucoside | 41.72 | 0.69 |
|  | HQ8 | MOL000378 | 7-O-methylisomucronulatol | 74.69 | 0.3 |
|  | HQ9 | MOL000379 | 9,10-dimethoxypterocarpan-3-O-β-D-glucoside | 36.74 | 0.92 |
|  | N1 | MOL000380 | (6aR,11aR)-9,10-dimethoxy-6a,11a-dihydro-6H-benzofurano[3,2-c]chromen-3-ol | 64.26 | 0.42 |
|  | HQ11 | MOL000387 | Bifendate | 31.1 | 0.67 |
|  | N2 | MOL000392 | Formononetin | 69.67 | 0.21 |
|  | HQ13 | MOL000398 | Isoflavanone | 109.99 | 0.3 |
|  | HQ14 | MOL000417 | Calycosin | 47.75 | 0.24 |
|  | B1 | MOL000422 | Kaempferol | 41.88 | 0.24 |
|  | HQ16 | MOL000433 | FA | 68.96 | 0.71 |
|  | HQ17 | MOL000438 | (3R)-3-(2-hydroxy-3,4-dimethoxyphenyl)chroman-7-ol | 67.67 | 0.26 |
|  | HQ18 | MOL000439 | Isomucronulatol-7,2'-di-O-glucosiole | 49.28 | 0.62 |
|  | HQ19 | MOL000442 | 1,7-Dihydroxy-3,9-dimethoxy pterocarpene | 39.05 | 0.48 |
|  | C1 | MOL000098 | Quercetin | 46.43 | 0.28 |
| Gardenia,Cape Jasmine (ZZ) | ZZ1 | MOL001406 | Crocetin | 35.3 | 0.26 |
|  | ZZ2 | MOL001663 | (4aS,6aR,6aS,6bR,8aR,10R,12aR,14bS)-10-hydroxy-2,2,6a,6b,9,9,12a-heptamethyl-1,3,4,5,6,6a,7,8,8a,10,11,12,13,14b-tetradecahydropicene-4a-carboxylic acid | 32.03 | 0.76 |
|  | ZZ3 | MOL001941 | Ammidin | 34.55 | 0.22 |
|  | ZZ4 | MOL004561 | Sudan III | 84.07 | 0.59 |
|  | C1 | MOL000098 | Quercetin | 46.43 | 0.28 |
|  | A1 | MOL000358 | Beta-sitosterol | 36.91 | 0.75 |
|  | B1 | MOL000422 | Kaempferol | 41.88 | 0.24 |
|  | S1 | MOL000449 | Stigmasterol | 43.83 | 0.76 |
|  | R1 | MOL001494 | Mandenol | 42 | 0.19 |
|  | ZZ10 | MOL001506 | Supraene | 33.55 | 0.42 |
|  | H1 | MOL001942 | Isoimperatorin | 45.46 | 0.23 |
|  | ZZ12 | MOL002883 | Ethyl oleate (NF) | 32.4 | 0.19 |
|  | ZZ13 | MOL003095 | 5-hydroxy-7-methoxy-2-(3,4,5-trimethoxyphenyl)chromone | 51.96 | 0.41 |
|  | ZZ14 | MOL007245 | 3-Methylkempferol | 60.16 | 0.26 |
|  | ZZ15 | MOL009038 | GBGB，Genipin 1-gentiobioside | 45.58 | 0.83 |
| Chinaberry (CLZ) | R1 | MOL001494 | Mandenol（Ethyl Linoleate） | 42 | 0.19 |
|  | CLZ2 | MOL001495 | Ethyl linolenate（9,12,15-Octadecatrienoic acid ethyl ester） | 46.1 | 0.2 |
|  | CLZ3 | MOL002045 | Stigmasterol | 43.41 | 0.76 |
|  | CLZ4 | MOL002047 | Melianone | 40.73 | 0.81 |
|  | CLZ5 | MOL002048 | Nimbolidin D | 30.38 | 0.53 |
|  | CLZ6 | MOL002053 | Nimbolin A | 32.11 | 0.34 |
|  | CLZ7 | MOL002056 | (E)-3-[(2S,3R)-2-(4-hydroxy-3-methoxy-phenyl)-7-methoxy-3-methylol-2,3-dihydrobenzofuran-5-yl]acrolein（(+)-Balanophonin） | 54.74 | 0.4 |
|  | CLZ8 | MOL002058 | 40957-99-1（Medioresinol） | 57.2 | 0.62 |
|  | C1 | MOL000098 | Quercetin | 46.43 | 0.28 |
| Safflower (HH) | I1 | MOL001771 | Poriferast-5-en-3beta-ol | 36.91 | 0.75 |
|  | HH2 | MOL002680 | Flavoxanthin | 60.41 | 0.56 |
|  | HH3 | MOL002694 | 4-[(E)-4-(3,5-dimethoxy-4-oxo-1-cyclohexa-2,5-dienylidene)but-2-enylidene]-2,6-dimethoxycyclohexa-2,5-dien-1-one | 48.47 | 0.36 |
|  | HH4 | MOL002695 | Lignan | 43.32 | 0.65 |
|  | HH5 | MOL002698 | Lupeol-palmitate | 33.98 | 0.32 |
|  | HH6 | MOL002706 | Phytoene | 39.56 | 0.5 |
|  | HH7 | MOL002707 | Phytofluene | 43.18 | 0.5 |
|  | HH8 | MOL002710 | Pyrethrin II | 48.36 | 0.35 |
|  | HH9 | MOL002712 | 6-Hydroxykaempferol | 62.13 | 0.27 |
|  | HH10 | MOL002714 | Baicalein | 33.52 | 0.21 |
|  | HH11 | MOL002717 | Qt_carthamone | 51.03 | 0.2 |
|  | HH12 | MOL002719 | 6-Hydroxynaringenin | 33.23 | 0.24 |
|  | HH13 | MOL002721 | Quercetagetin | 45.01 | 0.31 |
|  | HH14 | MOL002757 | 7,8-dimethyl-1H-pyrimido[5,6-g]quinoxaline-2,4-dione | 45.75 | 0.19 |
|  | HH15 | MOL002773 | Beta-carotene | 37.18 | 0.58 |
|  | J1 | MOL002776 | Baicalin | 40.12 | 0.75 |
|  | A1 | MOL000358 | Beta-sitosterol | 36.91 | 0.75 |
|  | B1 | MOL000422 | Kaempferol | 41.88 | 0.24 |
|  | S1 | MOL000449 | Stigmasterol | 43.83 | 0.76 |
|  | K1 | MOL000006 | Luteolin | 36.16 | 0.25 |
|  | T1 | MOL000953 | CLR | 37.87 | 0.68 |
|  | C1 | MOL000098 | Quercetin | 46.43 | 0.28 |
| Dwarf Lilyturf Tuber (MD) | MD1 | MOL000546 | Diosgenin | 80.88 |  |
|  | MD2 | MOL001788 | Adenine | 62.81 |  |
|  | MD3 | MOL007559 | Hordenine | 46.96 |  |
|  | MD4 | MOL000430 | Betaine | 40.92 |  |
|  | MD5 | MOL000114 | Vanillic Acid | 35.47 |  |
| Notoginseng (SQ) | R1 | MOL001494 | Mandenol | 42 | 0.19 |
|  | U1 | MOL001792 | DFV | 32.76 | 0.18 |
|  | SQ3 | MOL002879 | Diop | 43.59 | 0.39 |
|  | A1 | MOL000358 | Beta-sitosterol | 36.91 | 0.75 |
|  | S1 | MOL000449 | Stigmasterol | 43.83 | 0.76 |
|  | SQ6 | MOL005344 | Ginsenoside rh2 | 36.32 | 0.56 |
|  | SQ7 | MOL007475 | Ginsenoside f2 | 36.43 | 0.25 |
|  | C1 | MOL000098 | Quercetin | 46.43 | 0.28 |
| Terminalia chebula (KZ) | KZ1 | MOL001907 | ACar 18:0 |  |  |
|  | KZ2 | MOL001908 | 1,2,6-tri-O-galloyl-B-D-glucopyranose |  |  |
|  | KZ3 | MOL001909 | 1,6-bis-O-galloyl-beta-D-glucose |  |  |
|  | KZ4 | MOL001910 | 12-Deuteriododecanoic Acid |  |  |
|  | KZ5 | MOL001911 | 2,4-chebulyl-b-D-glucopyranose |  |  |
|  | KZ6 | MOL001912 | 23-O galloylpinfaenoic acid 28-O-β-D-glucopyranosyl ester |  |  |
|  | KZ7 | MOL001913 | 23-O-4′-epi-neochebuloylarjungenin |  |  |
|  | KZ8 | MOL001914 | 23-O-neochebuloylarjungenin 28-O-β–Dglycopyranosyl ester |  |  |
|  | KZ9 | MOL001915 | 3,4,6-tri-O-galloyl-D-glucose |  |  |
|  | KZ10 | MOL001916 | 3,4-DIHYDROXYBENZOIC ACID; protocatechuic acid |  |  |
|  | KZ11 | MOL001917 | Arjungenin |  |  |
|  | KZ12 | MOL001918 | Arjunic acid |  |  |
|  | KZ13 | MOL001919 | Brevifolin carboxylic acid |  |  |
|  | KZ14 | MOL001920 | C16H33 |  |  |
|  | KZ15 | MOL001921 | Casuarinin |  |  |
|  | KZ16 | MOL001922 | Chebulagic acid |  |  |
|  | KZ17 | MOL001923 | Chebulanin |  |  |
|  | KZ18 | MOL001924 | Chebulic acid |  |  |
|  | KZ19 | MOL001925 | Chebulinic acid |  |  |
|  | KZ20 | MOL001926 | Corilagin |  |  |
|  | W1 | MOL001002 | Ellagic acid |  |  |
|  | KZ22 | MOL001003 | Ethyl gallate |  |  |
|  | KZ23 | MOL001004 | Ferulic acid |  |  |
|  | KZ24 | MOL001906 | Gallic acid |  |  |
|  | KZ25 | MOL028007 | Galloflavin |  |  |
|  | KZ26 | MOL028008 | Hexadecane |  |  |
|  | KZ27 | MOL028009 | Lavendustin A |  |  |
|  | K1 | MOL000006 | Luteolin |  |  |
|  | KZ29 | MOL000007 | Methyl gallate |  |  |
|  | KZ30 | MOL000008 | Monobutyl phthalate |  |  |
|  | KZ31 | MOL000009 | Neochebulinic acid |  |  |
|  | KZ32 | MOL000010 | Pentagalloylglucose |  |  |
|  | KZ33 | MOL000011 | Punicalagin |  |  |
|  | KZ34 | MOL000012 | Stearate |  |  |
|  | KZ35 | MOL000013 | Stearic Acid |  |  |
|  | KZ36 | MOL000014 | Terchebulin |  |  |
|  | KZ37 | MOL000015 | Terflavin B |  |  |
| Rosewood Heart Wood (JX) | D1 | MOL001040 | (2R)-5,7-dihydroxy-2-(4-hydroxyphenyl)chroman-4-one | 42.36 | 0.21 |
|  | U1 | MOL001792 | DFV | 32.76 | 0.18 |
|  | JX3 | MOL000228 | (2R)-7-hydroxy-5-methoxy-2-phenylchroman-4-one | 55.23 | 0.2 |
|  | JX4 | MOL002565 | Medicarpin | 49.22 | 0.34 |
|  | JX5 | MOL002914 | Eriodyctiol (flavanone) | 41.35 | 0.24 |
|  | JX6 | MOL002938 | (3R)-4'-Methoxy-2',3,7-trihydroxyisoflavanone | 68.86 | 0.27 |
|  | JX7 | MOL002939 | (3R)-5'-Methoxyvestitol | 83.06 | 0.26 |
|  | JX8 | MOL002940 | (3R)-3-(2,3-dihydroxy-4-methoxyphenyl)-7-hydroxychroman-4-one | 52.06 | 0.27 |
|  | JX9 | MOL002941 | (3R)-3-(2,3-dihydroxy-4-methoxyphenyl)chroman-7,8-diol | 82.35 | 0.27 |
|  | JX10 | MOL002950 | (3R)-7,2',3'-trihydroxy-4-methoxyisoflavan | 69.65 | 0.24 |
|  | JX11 | MOL002957 | 9-O-Methylcoumestrol | 33.73 | 0.38 |
|  | JX12 | MOL002958 | 3'-Hydroxymelanettin | 30.69 | 0.27 |
|  | JX13 | MOL002959 | 3'-Methoxydaidzein | 48.57 | 0.24 |
|  | JX14 | MOL002961 | (-)-Vestitol | 70.29 | 0.21 |
|  | JX15 | MOL002962 | (3S)-7-hydroxy-3-(2,3,4-trimethoxyphenyl)chroman-4-one | 48.23 | 0.33 |
|  | JX16 | MOL002963 | 4',5',7-trimethyl-3-methoxyflavone | 40.66 | 0.25 |
|  | JX17 | MOL002966 | Dalbergin | 78.18 | 0.2 |
|  | JX18 | MOL002967 | 7-hydroxy-4'-methoxy-2',5'-dioxo-4-[(3R)-2',7-dihydroxy-4'-methoxyisoflavan-5'-yl]isoflavane | 34.78 | 0.7 |
|  | JX19 | MOL002973 | Bowdichione | 55.78 | 0.28 |
|  | JX20 | MOL002975 | Butin | 69.94 | 0.21 |
|  | JX21 | MOL002981 | Duartin | 70.63 | 0.34 |
|  | JX22 | MOL002982 | (3R,4R)-3',7-dihydroxy-2',4'-dimethoxy-4-[(2S)-4',5,7-trihydroxyflavanone-6-yl]isoflavan | 33.96 | 0.63 |
|  | JX23 | MOL002985 | Isoduartin | 74.11 | 0.34 |
|  | JX24 | MOL002989 | 4-Hydroxyhomopterocarpin | 48.41 | 0.43 |
|  | JX25 | MOL002990 | (6aR,11aR)-3,9,10-trimethoxy-6a,11a-dihydro-6H-benzofurano[3,2-c]chromen-4-ol | 66.86 | 0.53 |
|  | JX26 | MOL002991 | (6aR,11aR)-3,9-dimethoxy-6a,11a-dihydro-6H-benzofurano[3,2-c]chromene-4,10-diol | 38.96 | 0.48 |
|  | JX27 | MOL002996 | Odoricarpin | 55.02 | 0.53 |
|  | JX28 | MOL002997 | 3-(2-hydroxy-3,4-dimethoxyphenyl)-2H-chromen-7-ol | 86.18 | 0.27 |
|  | JX29 | MOL002999 | Sativanone | 85.63 | 0.27 |
|  | JX30 | MOL003000 | Stevein | 36.54 | 0.24 |
|  | JX31 | MOL003001 | Vestitone | 52.83 | 0.24 |
|  | JX32 | MOL003002 | Violanone | 80.24 | 0.3 |
|  | JX33 | MOL003003 | Xenognosin B | 72.71 | 0.24 |
|  | A1 | MOL000358 | Beta-sitosterol | 36.91 | 0.75 |
|  | M1 | MOL000359 | Sitosterol | 36.91 | 0.75 |
|  | N1 | MOL000380 | (6aR,11aR)-9,10-dimethoxy-6a,11a-dihydro-6H-benzofurano[3,2-c]chromen-3-ol | 64.26 | 0.42 |
|  | N2 | MOL000392 | Formononetin | 69.67 | 0.21 |
| Sandalwood (TX) | O1 | MOL000354 | Isorhamnetin | 49.6 | 0.31 |
|  | K1 | MOL000006 | Luteolin | 36.16 | 0.25 |
|  | TX3 | MOL002322 | Isovitexin | 31.29 | 0.72 |
| Bovine pericardium (NX) | NX1 |  | 6-Methyl-2-aminobenzoxazol |  |  |
|  | NX2 |  | Reticuline |  |  |
| Aucklandiae Radix (MX) | MX1 | MOL010813 | Benzo[a]carbazole | 35.22 | 0.22 |
|  | MX2 | MOL010828 | Cynaropicrin | 67.5 | 0.38 |
|  | MX3 | MOL010839 | Lappadilactone | 38.56 | 0.73 |
|  | P1 | MOL000211 | Mairin | 55.38 | 0.78 |
|  | M1 | MOL000359 | Sitosterol | 36.91 | 0.75 |
|  | S1 | MOL000449 | Stigmasterol | 43.83 | 0.76 |
| Rhubarb (DH) | DH1 | MOL002235 | EUPATIN | 50.8 | 0.41 |
|  | DH2 | MOL002251 | Mutatochrome | 48.64 | 0.61 |
|  | DH3 | MOL002259 | Physciondiglucoside | 41.65 | 0.63 |
|  | DH4 | MOL002260 | Procyanidin B-5,3'-O-gallate | 31.99 | 0.32 |
|  | DH5 | MOL002268 | Rhein | 47.07 | 0.28 |
|  | DH6 | MOL002276 | Sennoside E_qt | 50.69 | 0.61 |
|  | DH7 | MOL002280 | Torachrysone-8-O-beta-D-(6'-oxayl)-glucoside | 43.02 | 0.74 |
|  | DH8 | MOL002281 | Toralactone | 46.46 | 0.24 |
|  | DH9 | MOL002288 | Emodin-1-O-beta-D-glucopyranoside | 44.81 | 0.8 |
|  | DH10 | MOL002293 | Sennoside D_qt | 61.06 | 0.61 |
|  | DH11 | MOL002297 | Daucosterol_qt | 35.89 | 0.7 |
|  | DH12 | MOL002303 | Palmidin A | 32.45 | 0.65 |
|  | A1 | MOL000358 | Beta-sitosterol | 36.91 | 0.75 |
|  | DH14 | MOL000471 | Aloe-emodin | 83.38 | 0.24 |
|  | DH15 | MOL000554 | Gallic acid-3-O-(6'-O-galloyl)-glucoside | 30.25 | 0.67 |
|  | F1 | MOL000096 | (-)-catechin | 49.68 | 0.24 |
| Ophiopogon japonicus (MT) | MT1 | MOL010799 | Ariskanin A | 109.51 | 0.4 |
|  | MT2 | MOL010803 | Aristolochic acid A | 62.71 | 0.55 |
|  | MT3 | MOL010804 | Aristolochic acidⅡ | 37.56 | 0.45 |
|  | I1 | MOL001771 | Poriferast-5-en-3beta-ol | 36.91 | 0.75 |
|  | Q1 | MOL000296 | Hederagenin | 36.91 | 0.75 |
|  | A1 | MOL000358 | Beta-sitosterol | 36.91 | 0.75 |
|  | S1 | MOL000449 | Stigmasterol | 43.83 | 0.76 |
|  | MT8 | MOL000763 | Aristoloside_qt | 40.32 | 0.6 |
| Long Pepper (BB) | BB1 | MOL001555 | ZINC03996196 | 52.35 | 0.62 |
|  | BB2 | MOL001558 | Sesamin | 56.55 | 0.83 |
|  | BB3 | MOL001559 | Piperlonguminine | 30.71 | 0.18 |
|  | BB4 | MOL001560 | Pipernonaline | 51.32 | 0.41 |
|  | BB5 | MOL001561 | Dehydropipernonaline | 47.73 | 0.41 |
|  | BB6 | MOL001586 | N-(2,5-dimethoxyphenyl)-4-methoxybenzamide | 60.7 | 0.18 |
|  | BB7 | MOL001588 | N-isobutyleicosa-2(E),4(E),8(Z)-trienamide | 44.48 | 0.32 |
|  | BB8 | MOL001589 | N-Isobutyl-2,4-icosadienamide | 38.86 | 0.32 |
|  | BB9 | MOL001592 | Piperine | 42.52 | 0.23 |
|  | BB10 | MOL001594 | Pisatin | 88.05 | 0.64 |
|  | L1 | MOL001601 | 1,2,5,6-tetrahydrotanshinone | 38.75 | 0.36 |
|  | BB12 | MOL001607 | ZINC03982454 | 36.91 | 0.76 |
|  | BB13 | MOL001610 | Sylvatine | 44 | 0.51 |
|  | BB14 | MOL001614 | (E,E,E)-11-(1,3-Benzodioxol-5-yl)-N-(2-methylpropyl)-2,4,10-undecatrienenamide | 42.72 | 0.43 |
|  | BB15 | MOL001616 | 1-[1-oxo-9(3, 4-methylenedioxyphenyl)-2E,8E-nonadienyl] pyrrolidine | 49.43 | 0.36 |
| Artificial Moschus (SX) | SX1 | MOL002476 | 2,6-Nonamethylene Pyridine | 68.94 |  |
|  | SX2 | MOL004480 | Acetic Acid | 47.87 |  |
|  | SX3 | MOL000737 | Morin | 46.23 |  |
|  | SX4 | MOL007330 | Menthol | 43.31 |  |
|  | V1 | MOL008845 | Deoxycholic Acid | 40.72 |  |
|  | SX6 | MOL003790 | Androstane | 32.50 |  |
|  | SX7 | MOL000991 | Cinnamaldehyde | 31.99 |  |
| Artificial Bezoa (NH) | NH3 | MOL008846 | ZINC01280365 | 46.38 | 0.49 |
|  | V1 | MOL008845 | Deoxycholic Acid | 40.72 | 0.68 |
|  | T1 | MOL000953 | CLR (Cholesterol) | 37.87 | 0.68 |
|  | NH9 | MOL008839 | Methyl desoxycholate | 34.63 | 0.73 |
|  | NH11 | MOL008838 | methyl (4R)-4-[(3R,5S,7S,8R,9S,10S,12S,13R,14S,17R)-3,7,12-trihydroxy-10,13-dimethyl-2,3,4,5,6,7,8,9,11,12,14,15,16,17-tetradecahydro-1H-cyclopenta[a]phenanthren-17-yl]pentanoate | 32.32 | 0.76 |
